# Functional enrichment of gut microbiome by early supplementation of *Bacillus* based probiotic in cage free hens: a field study

**DOI:** 10.1186/s42523-021-00112-5

**Published:** 2021-07-27

**Authors:** Samiullah Khan, Kapil K. Chousalkar

**Affiliations:** School of Animal and Veterinary Sciences, The University of Adelaide, Roseworthy, South Australia 5371 Australia

**Keywords:** Gut microbiome, Free-range chicken, Probiotic, Microbial diversity, Microbial abundance

## Abstract

**Background:**

The chicken gut microbiota passes through different stages of maturation; therefore, strengthening it with well characterised probiotics increases its resilience required for optimum gut health and wellbeing. However, there is limited information on the interaction of *Bacillus* based probiotics with gut microbial community members in cage free laying chickens both in rearing and production phases of life. In the current study, we investigated the changes in the gut microbiome of free range hens in the field after *Bacillus* based probiotic supplementation.

**Results:**

Overall, at phylum level, probiotic supplementation increased the populations of Bacteroidetes and Proteobacteria mainly at the expense of Firmicutes. The population of Bacteroidetes significantly increased during the production as compared to the rearing phase, and its higher population in the probiotic-supplemented chickens reflects the positive role of *Bacillus* based probiotic in gut health. Core differences in the beta diversity suggest that probiotic supplementation decreased microbial compositionality. The non-significant difference in alpha diversity between the probiotic and control chickens showed that the composition of community structure did not change. No *Salmonella* spp. were isolated from the probiotic supplemented birds. Egg internal quality was significantly higher, while egg production and body weight did not differ. Functional prediction data showed that probiotic supplementation enriched metabolic pathways, such as vitamin B6 metabolism, phenylpropanoid biosynthesis, monobactam biosynthesis, RNA degradation, retinol metabolism, pantothenate and CoA biosynthesis, *phosphonate and phosphinate metabolism, AMPK signaling pathway, cationic antimicrobial peptide (CAMP) resistance and tyrosine metabolism.*

**Conclusions:**

Overall, age was the main factor affecting the composition and diversity of gut microbiota, where probiotic supplementation improved the abundance of many useful candidates in the gut microbial communities. The generated baseline data in the current study highlights the importance of the continuous use of *Bacillus* based probiotic for optimum gut health and production.

**Supplementary Information:**

The online version contains supplementary material available at 10.1186/s42523-021-00112-5.

## Background

In a healthy state, the assemblage of viruses, bacteria, archaea, and fungi forms a host microbiome that can mediate biological functions ranging from energy metabolism to immune response biomolecules production [1–4]. A stable consortium of commensal gut microbial communities is associated with pathogen exclusion [5], as reduced diversity has been shown to increase the risk of infection [6, 7]. The 16S rRNA metagenomics data have disclosed differences in community membership of different age groups, differences that contribute to pathophysiological functions in a host. For example, metagenomics data from murine, human and chicken models indicate that the host-microbiota cross-talk influences response to cell injury [8], affects energy balance [9], supports the synthesis and biotransformation of isoprenoids, vitamins, xenobiotics, amino acids and glycans [10], provides colonisation resistance against pathogens [6, 11] and influences the maturation of immune system [12]. In the host, the fermentation process is mainly achieved by microbiota through the production of gut metabolites that include indole and its derivatives, linoleic acids, tryptamine, short-chain fatty acids (SCFAs) and vitamins [13, 14].

In layer chickens, gut microbiota influences performance and resistance to pathogens, such as *Salmonella* and *Campylobacter*. For example, an increased abundance of *Faecalibacterium* resulted in clearing *Salmonella* Typhimurium from the gut [6]. Higher abundance levels of Fusobacteria and Bacteroides were associated with increased egg production [15]. Gut health is maintained partly by the resident gut microbiota that provides the first line of resistance against pathogen colonisation. However, the composition and diversity of gut microbiota vary with genotype [16], rearing conditions, age [17, 18] and stress factors [19–21]. Affected by body weight, certain species of *Lactobacillus*, and *Lactococcus lactis* and *Bacillus thermoamylovorans* were significantly higher in abundance in low body-weight laying hens, suggesting the role of these bacteria in body composition of the host [22]. Assessing the age effect on gut microbiota composition, compared to 8 weeks of chickens’ age, Bacteroidetes and Firmicutes were more abundant in the gut of 30 week laying hens [17]. A comparative analysis of the gut microbiota of cage and free-range production systems showed a higher abundance of Bacteroidetes in free-range laying hens [23]. This suggests that a range of factors affect gut microbiota; therefore, strategies that help in enhancing gut health will result in lower colonisation of the gut by pathogens and improve hens’ performance.

One way to improve the diversity and composition of gut microbial communities is to supplement the diet with probiotics [13]. Probiotics are viable bacteria that maintain gut health through the production of organic acids [24], prime the immune system [25, 26] and help in the saturation of enterocytes [27] for pathogen colonisation resistance. Bacteria such as *Lactobacillus*, *Bacillus*, *Bifidobacterium* and *Streptococcus* are used for probiotics formulation that are commercially available for poultry industry. Currently, there are limited reports on the development and maturation of gut microbiota in cage free/free-range laying chickens in the field [17, 28, 29]. However, the microbiota of free range production is more diverse than cage chickens [17]. Finding microbial communities associated with the layer performance, and whether the *Bacillus* based probiotic plays a role in improving the diversity and composition of gut microbiota in free-range laying chickens will help in devising strategies for improving gut health for product safety. The optimised probiotic can be used for the control of food safety pathogens, such as *Listeria*, *Clostridium*, *Salmonella* and *Campylobacter*. Therefore, we hypothesised that if used from the day of a hatch in the field, *Bacillus* based probiotic will improve the composition of gut microbiota, enhance egg quality and reduce gut pathogen colonisation in cage free hens in the field. The chosen *Bacillus* based probiotic is commercially available as a premix for poultry feed. The main objective of this study was to test the effects of *Bacillus* based probiotic on gut health and layer performance in free-range production system.

## Methods

### Animal ethics and experimental design

The experimental work was approved by the Animal Ethics Committee at The University of Adelaide under approval number S-2019-109. Faecal swab collections were performed as per standard operative procedures approved by the Animal Ethics Committee.

A commercial farm was selected based on the willingness of the farm manager and the farm set up that was appropriate for this study. Prior to placing the chicks, environmental swabs (*n* = 40) were collected from empty rearing sheds (labelled as control and probiotic supplemented) before and after the clean-up procedures to determine the contamination level of *Salmonella* spp. (if any). The experimental flock (Hyline Brown) was distributed into 2 rearing sheds on a pullet-rearing farm from day 1 of placement with 10,860 and 10,457 chicks in the control and probiotic supplemented sheds, respectively. The stocking density was 30 birds/m^2^. One shed acted as a control, while chicks in the other shed received a premix of *Bacillus* based probiotic at a rate of 1 g/kg of feed from day 1 until the termination of the experiment (week 36 of flock age). The commercially available probiotic was composed of *Bacillus subtilis* DSM 32324, *Bacillus subtilis* DSM 32325 and *Bacillus amyloliquifaciens* DSM 25840. This probiotic was chosen as its continuous supplementation in a pen trial resulted in the overall lower shedding level of *Salmonella* Typhimurium [6]. Birds in both the sheds received vaccines against coccidiosis, infectious bronchitis, avian encephalomyelitis, New Castle disease, egg drop syndrome, infectious laryngotracheitis and fowl cholera. The pullets were raised on a concrete floor and they received chick starter feed from 1 to 6 weeks, a grower from 7 to 12 weeks, a developer from 13 to 14 weeks, pre-lay from 15 to 17 weeks and peak lay diet from week 18 onwards (Additional file 1; Tables S1–5). Prior to shifting pullets to a production farm, the production sheds were swabbed (*n* = 40) to determine the contamination level of *Salmonella* spp. (if any). The pullets were shifted to free-range production sheds (control and probiotic supplemented) at 16-week of flock age. The distance between the rearing and production farms is approximately 30 km. Fresh faeces (*n* = 20) for DNA extraction, faecal swabs (*n* = 20) and environmental dust swabs (*n* = 10) for *Salmonella* isolation from each treatment group at each sampling time point were collected at day 1 (meconium samples), 5, 21, week 6, 12, 16 (Day 1 and 5 after shifting), 18, 24, 30 and 36 of flock age. Therefore, for 11 sampling time-points, a total of 440 faecal DNA samples, 440 faecal swab samples and 220 environmental dust swab samples were processed during this study. Once in lay, eggs (*n* = 30) from each treatment group were collected at week 24, 30 and 36 of flock age and processed for egg quality measurements. Faecal swab samples were collected in 4 mL buffered peptone water (BPW, ThermoFisher Scientific, Australia), while environmental swabs (Whirl–Pak “Speci-Sponge, ThermoFisher Scientific, Australia) were soaked in 20 mL BPW and individual swabs were dragged to cover at least 1 m^2^ area in the shed including exhaust fans and covering boards of nest boxes. Shoe covers from each shed were soaked in 150 mL BPW and processed for *Salmonella* isolation.

### Processing of faecal and environmental swabs for *Salmonella* isolation

The collected samples were processed for the isolation of *Salmonella* spp. as outlined in Additional file 1. Briefly, the faecal and environmental dust swab samples were incubated overnight and then enriched in Rappaport-Vassiliadis soya peptone (RVS) broth for the selective growth of *Salmonella*. The RVS overnight incubated samples were streaked on brilliance *Salmonella* and xylose lysine deoxycholate agar for the “YES” or “NO” confirmation of *Salmonella*. A miniaturized most probable number (mMPN) method was used to semi-quantify the load of *Salmonella* from the confirmed positive samples. Confirmed *Salmonella* isolates were further processed for traditional PCR using an invasion (invA) and TSR3 genes specific primers.

### Faecal DNA extraction and *16S rRNA* sequencing

Total DNA from the collected faeces was extracted following a modified protocol of QIAamp FAST DNA Mini Kit (Additional file 1). The undiluted DNA samples (*n* = 440; 20 samples per treatment group at each sampling for a total of 11 time-points) were sequenced by the Ramaciotti Centre for Genomics (University of New South Wales, Australia) for *16S rRNA* metagenome sequencing and subsequent data analysis for the generation of operational taxonomic units (OTUs) table. For microbial profiling, the hypervariable region (V3-V4) of the *16S rRNA* gene was sequenced using a barcoded primer pair (341F: 5′-CCTACGGGNGGCWGCAG-3′; 805R: 5′-GACTACHVGGGTATCTAATCC-3′).

### *16S rRNA* library preparation and Illumina sequencing

For individual faecal DNA samples, the *16S rRNA* library was prepared by a barcoding PCR in a 25 μL final reaction volume that contained 12.5 μL of KAPA HiFi HotStart Readymix PCR buffer (Kapa Biosystems), 9.5 μL of PCR grade water, 1 μL of each of the barcoded forward and reverse primers and 1 μL of faecal DNA template. The cycling conditions in SimpliAmp Thermal Cycler (Applied Biosystems) were: initial denaturation at 95 °C for 3 min, denaturation at 95 °C for 30 s, annealing at 55 °C for 30 s and elongation at 72 °C for 30 s for a total of 35 cycles. A final elongation at 72 °C for 5 min was included at the end of the cycles. The PCR amplicons were normalised and pooled using the SequalPrep Normalization Plate Kit (ThermoFisher Scientific, Australia) as per the manufacturer’s guidelines. The PCR library was purified using AxyPrep Mag PCR Clean-Up Kit (Fisher Biotec, Australia) as per the manufacturer’s protocol. The quality and concentration of the pooled library were assessed by Qubit, and the library size was estimated on an Agilent 2200 TapeStation (Integrated Science, Australia). From the pooled library, primer-dimer was removed by the Agencourt AMPure XP Bead Clean-up kit. The pooled library was sequenced on Illumina MiSeq using the MiSeq Reagent Kit v3 with a 2 × 300 bp run format as per the manufacturer’s protocol. For the MiSeq runs, custom primers were added to the reagent cartridge for Read1, Index and Read2.

### Microbial community data analysis and statistical tests

Raw sequencing reads (Fastq files) were processed with the OTUreporter v1.0.1-beta (5576d57) pipeline base on mothur (v1.39.5) [30, 31]. Briefly, the reads were quality filtered and assigned to their respective samples. Sequences were trimmed according to the MiSeq SOP [31] and only those with a length between 100 and 473 bp were retained, while longer than 8 bp homopolymer containing sequences were removed. For chimera removal, a chimera.vsearch script in mothur was used [32]. The sequences were aligned and classified against the SILVA reference alignment (v132) [33] and non-bacterial lineages not targeted by the barcoded primer pair (i.e. unknown, mitochondria, archaea, chloroplast, mitochondria and eukaryote) were removed. Sequences were grouped into OTUs based on 97% similarity using the OptiClust algorithm [34] and subsampled based on the sample with the lowest number of reads (*n* = 11,092). Sequencing error was assessed using the NO SEQ ERROR TEXT as a control in each run.

The OTU data were analysed in Calypso v 8.84 that functions based on R packages vegan [35] for microbiota phylogenetic analysis of community composition, abundance and diversity by taking probiotic supplementation and flock age as independent variables. In the Calypso, the OTU data were normalised using cumulative-sum scaling (CSS) and expressed as log_2_ relative abundance to account for the non-normal distribution of taxonomic counts data. In the CSS normalisation, raw counts are divided by the cumulative sum of counts up to a percentile determined using a data-driven approach [36]. Genus/Phylum abundance was determined using one-way ANOVA. Alpha diversity was calculated using the Shannon index that takes into account the richness and evenness of microbial communities. Beta diversity was calculated using ANOSIM based on Bray-Curtis dissimilarity matrices. Redundancy analysis (RDA+) was used for measuring variation in structural composition of the microbial community. For RDA+ analysis, both the control and probiotic supplemented groups were taken together without applying any corrections. To understand the effect of rearing phases and laying effects, data were also analysed for diversity analysis between the control and probiotic treatment groups based on rearing (Day 1 to Week 12 samples) and production (Week 16 to Week 36 samples) phases, pre-lay (Week 16 and Week 18 samples) and early-lay (Week 24 and Week 30 samples) periods.

### Functional predictions of metabolic pathways through *16S rRNA* data

The workflow of Tax4Fun2 [37] was used to predict the metabolic pathways of faecal microbiota. Differentially abundant features were identified using Welch’s t-test inbuilt in the statistical analysis of taxonomic and functional profile (STAMP) software [38], where features were filtered using *q value* > 0.05, leaving only the significant features to be visualised. In STAMP, Benjamini-Hochberg FDR was used for multiple test correction.

### Egg quality measurements

The collected eggs were processed for measuring egg weight, shell weight, shell thickness, albumen height, Haugh Unit and yolk colour following a previously described method [39]. Briefly, Technical Supplies and Services (TSS, UK) QCH albumen height gauge was used for albumen height measurement, while yolk colour was measured by DSM Yolk Colour fan (scale 1–16). Shell thickness was measured by Mitutoyo Dial Comparator Gauge Model 2109–10 (Kawasaki, Japan). Haugh Unit (HU) was measured from the egg weight and albumen height by using the following equation [40]:

HU = 100 * log_10_ (HT - 1.7 * EW^0.37 + 7.6); where HT is albumen height (mm) and EW is egg weight (g).

In addition, at the farm, egg production was recorded daily, while body weight of 100 birds from each shed was recorded weekly. Where appropriate, egg quality data were analysed in StatView v5.0.1.0 with one- or two- way ANOVA. Level of significance was established at protected least significant difference (PLSD) < 0.05. Flock performance data (body weight, lay rate, egg weight and FCR) were visualised in Excel Workbook.

## Results

### Metagenome data quality assessment and overall gut microbiota landscape

The sequence data assessment showed that out of 440 faecal DNA samples, 8 samples (1.81%) generated less than 10,000 reads per sample after passing the QC and chimera removal, and were therefore discarded from the downstream analysis (Additional file 2). Out of the 8 discarded samples, 7 samples were from day 1 meconium, which usually contains very little microbiota. After subsampling, the OTU coverage was ≥97%. The slope of the rarefaction curve indicated that the sequenced data covered most of the microbial communities associated with faeces (Additional file 3: Fig. S1 a, b). Therefore, the quality evaluation steps showed that the data were robust and suitable for faecal microbiota composition and diversity analyses. The OTU table (in mothur format) and the metadata file (CSV format) are included with the manuscript for reproducibility of this study (Additional files 4 and 5).

### Phylogenetic variation in faecal microbiome affected by probiotic supplementation and flock age

**Fig. 1:**
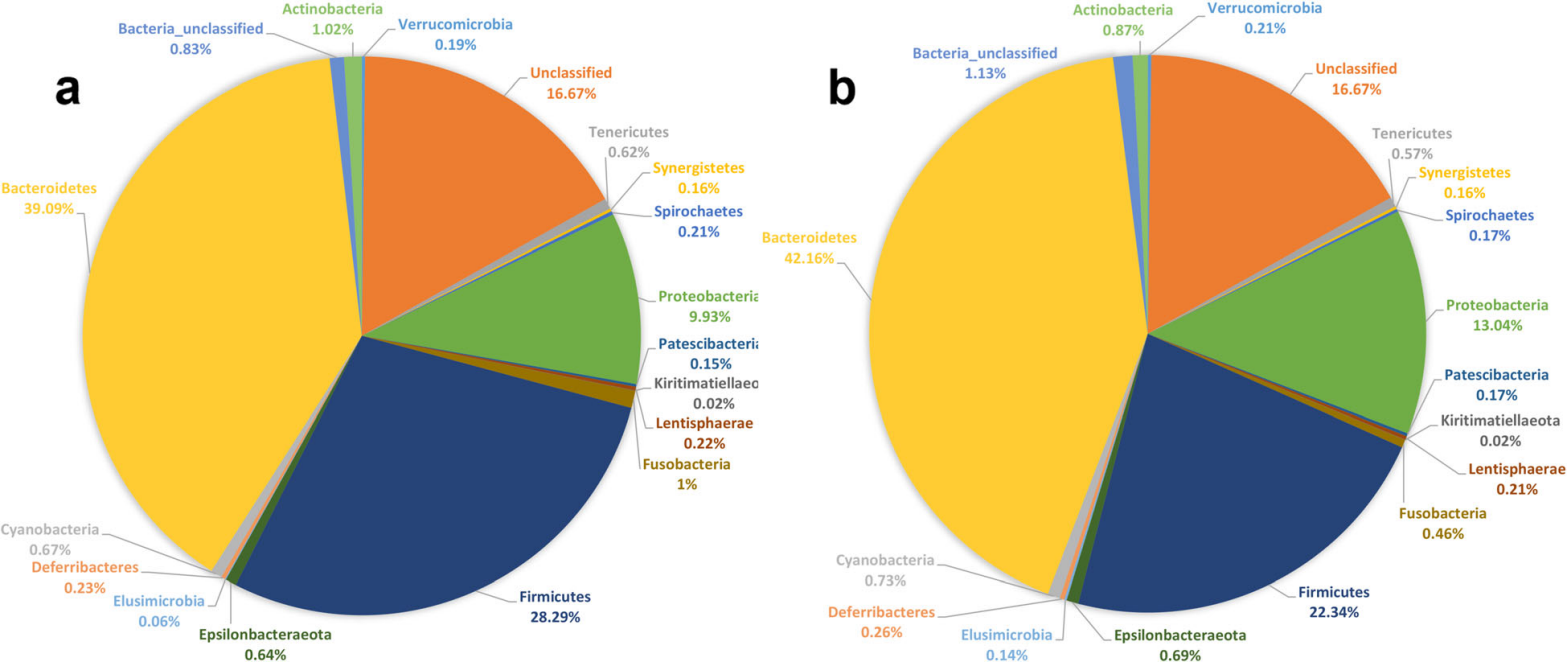
Overall gut microbiota composition at the phylum level affected by probiotic supplementation. a). Microbial abundance (%) in the control flock. b). Microbial abundance (%) in the probiotic supplemented flock. For direct comparison between the control and probiotic supplemented groups, data obtained from the samples collected between day 1 and week 36 of flock age were analysed as a pool. For percent calculation of microbial abundance at phylum level, total sum scaling normalised but untransformed data obtained from Calypso software were visualised in Excel 2016 and the panel graphs were prepared in Graphpad prism v. 8.0.0

Comprehensively, the gut microbiota of the control and probiotic supplemented flocks was composed of 15 known bacterial phyla out of which Bacteroidetes, Firmicutes and Proteobacteria were the most abundant (Additional file 6: Fig. S2). Overall, the abundance of Bacteroidetes, Firmicutes and Proteobacteria accounted for 44.45, 25.43 and 11.56%, respectively. Known phyla of bacteria that accounted for less than 1% of the total population included Actinobacteria (0.94%), Fusobacteria (0.73%), Epsilonbacteraeota (0.67%), Tenericutes (0.59%), Deferribacteres (0.25%), Lentisphaerae (0.21%), Verrucomicrobia (0.19%), Spirochaetes (0.18%), Synergistetes (0.16%), Patescibacteria (0.16%), Elusimicrobia (0.11%) and Kiritimatiellaeota (0.02%). Interestingly, probiotic supplementation significantly (*P* < 0.0001) decreased the overall abundance levels (%) of Firmicutes and Spirochaetes and increased (P < 0.0001) Elusimicrobia (Fig. 1 a, b). Although the percentage for Cyanobacteria was calculated while using the Calypso software, it is not considered as a part of the normal gut microbiota as they are photosynthetic and their presence in gut samples is assumed to be derived from ingested cyanobacterial cells or chloroplast.

**Fig. 2:**
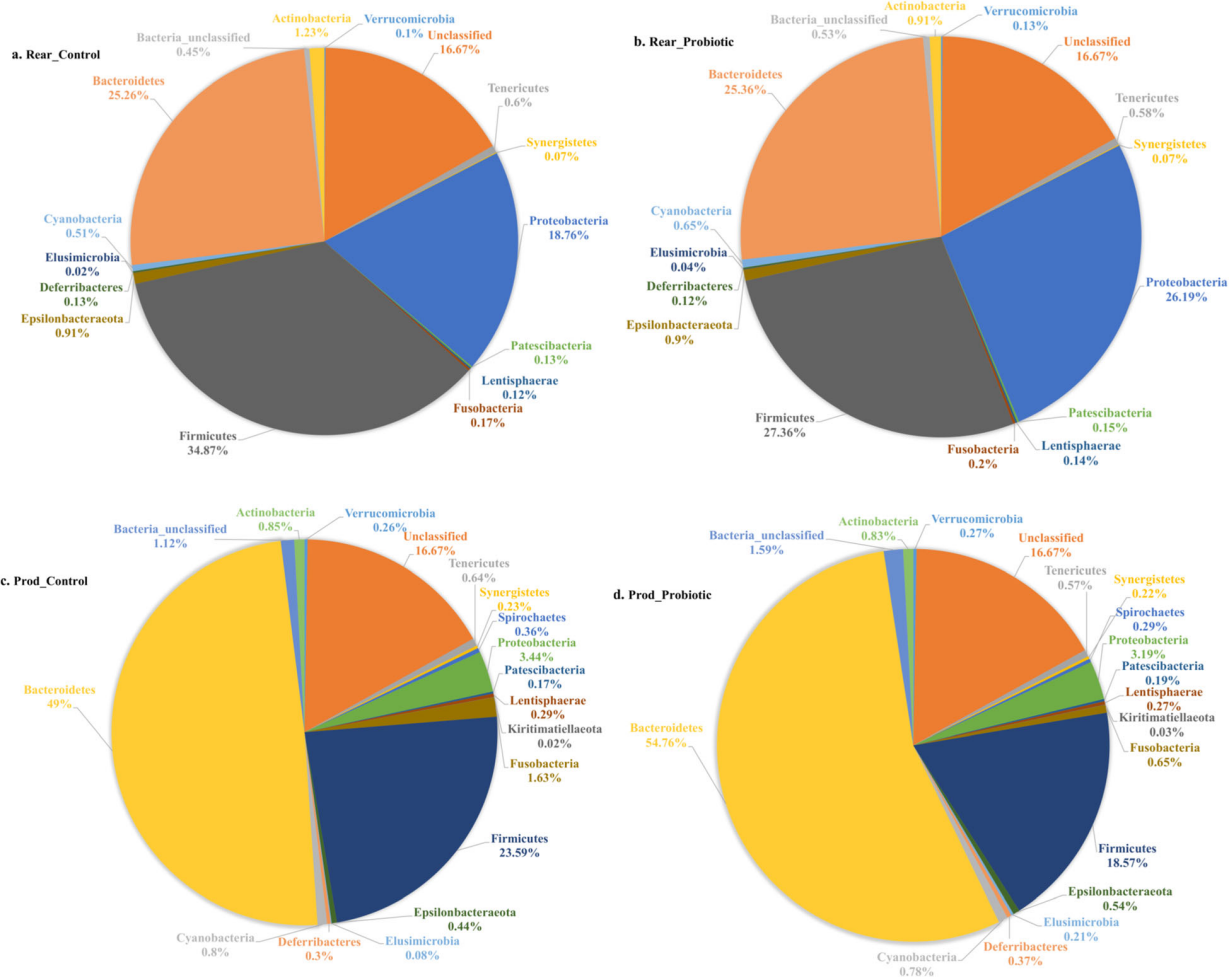
Overall gut microbiota composition at phylum level affected by probiotic supplementation in rearing and production phases of hens. **a**). Microbial abundance (%) of the control flock (Rear_Control) in the rearing phase. **b**). Microbial abundance (%) of the probiotic flock (Rear_Probiotic) in the rearing phase. **c**). Microbial abundance (%) of the control flock (Prod_Control) in the production phase. **d**). Microbial abundance (%) of the probiotic flock (Prod_Probiotic) in the production phase. For percent calculation of microbial abundance at the phylum level, total sum scaling normalised but untransformed data obtained from Calypso software were visualised in Excel 2016 and the panel graphs were prepared in Graphpad prism v. 8.0.0

The commercial layer life cycle is mainly comprised of rearing and production phases, where the rearing period is mainly up to 16 weeks of age and the chickens are kept on dirt or concrete floor. Assessing the effects of the probiotic supplementation in the rearing phase, the data showed that the abundance of Proteobacteria significantly increased while Firmicutes decreased (Fig. 2 a, b). In the production phase, in the probiotic supplemented flock, the abundance of Firmicutes remained lower, while Bacteroidetes increased compared with the control flock (Fig. 2 c, d).

**Fig. 3:**
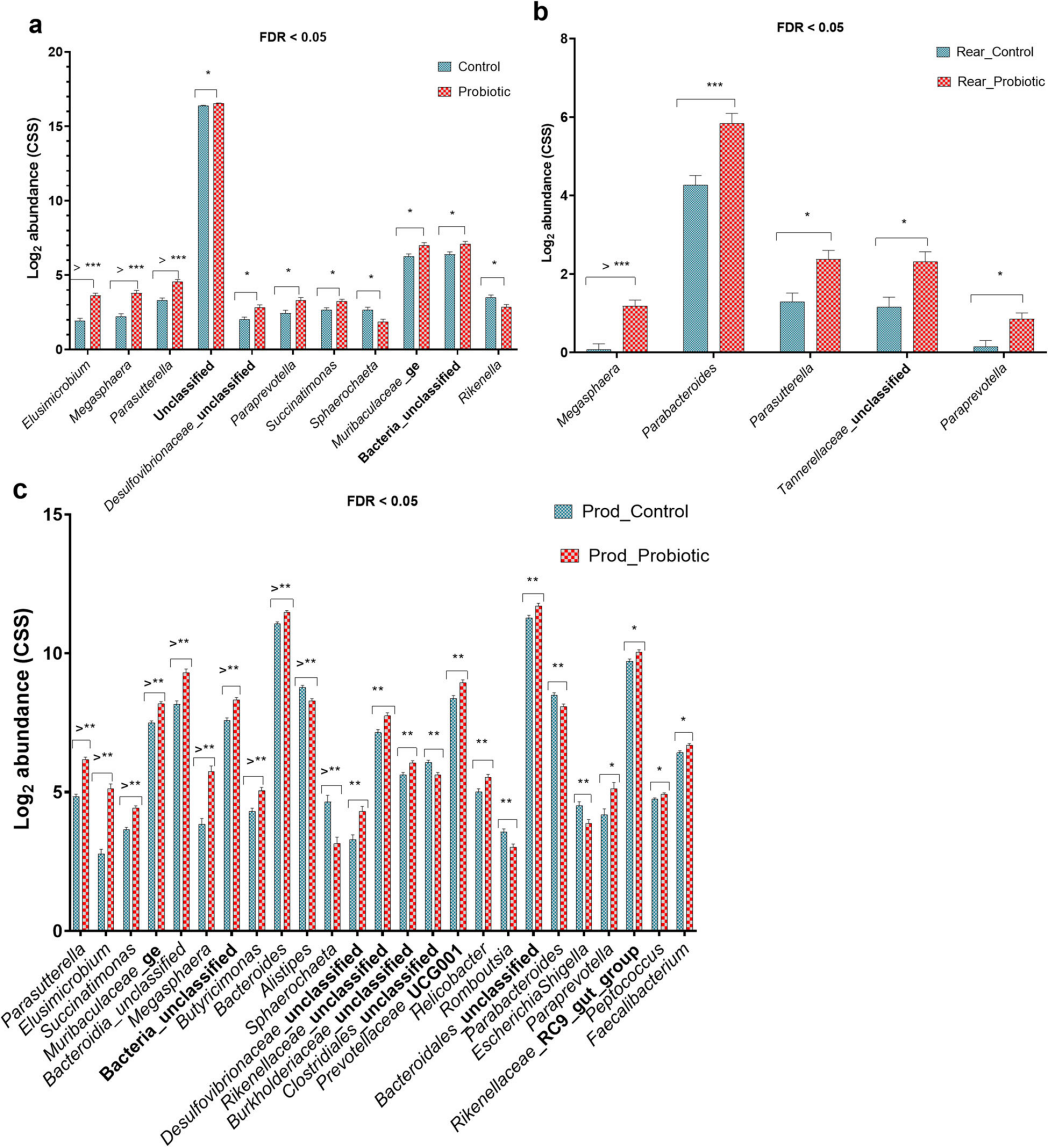
The abundance of gut microbiota at the genus level is affected by probiotic supplementation. **a**). Overall relative abundance levels of microbial communities affected by probiotic. **b**). Relative abundance levels of microbial communities in the rearing phase of layer between the control (Rear_Control) and probiotic supplemented (Rear_Probiotic) flocks. **c**). Relative abundance levels of microbial communities in the early production phase of layer between the control (Prod_Control) and probiotic supplemented (Prod_Probiotic) flocks. Relative abundance levels (in log_2_ cumulative sum scaling) was calculated in Calypso software and the data were visualised in GraphPad Prism v.8.0.0. Asterisks (*, ** and ***) show *P* values at 0.01, 0.001 and 0.0001, respectively

Probiotic supplementation increased the abundance of numerous microbial communities at the genus level (Fig. 3). Overall, there was a significantly higher abundance of *Elusimicrobium*, *Megasphaera*, *Parasutterella*, *Desulfovibrionaceae*_unclassified, *Paraprevotella*, *Succinatimonas*, Bacteria_unclassified and *Muribaculaceae*_ge in the probiotic compared with the control flock (Fig. 3 a). Probiotic supplementation reduced the abundance levels of *Sphaerochaeta* and *Rikenella*. The effects of probiotic supplementation were more prominent in terms of affecting the abundance of multiple genera in the production than the rearing phase of life (Fig. 3 b, c). In the rearing phase, probiotic supplementation increased the abundance levels of *Megasphaera*, *Parabacteroides*, *Parasutterella*, *Tannerellaceae*_unclassified and *Paraprevotella* (Fig. 3 b), while in the production phase, in addition to these, probiotic supplementation increased the abundance levels of a range of microbial communities including *Elusimicrobium*, *Butyricimonas*, *Bacteroides*, *Peptococcus* and *Faecalibacterium* (Fig. 3 c). However, the abundance of *Tannerellaceae*_unclassified was not significantly altered in the production phase of life. In the production phase, probiotic supplementation resulted in the decreased abundance of microbial communities, such as *Alistipes*, *Sphaerochaeta* and *Romboutsia*.

**Fig. 4:**
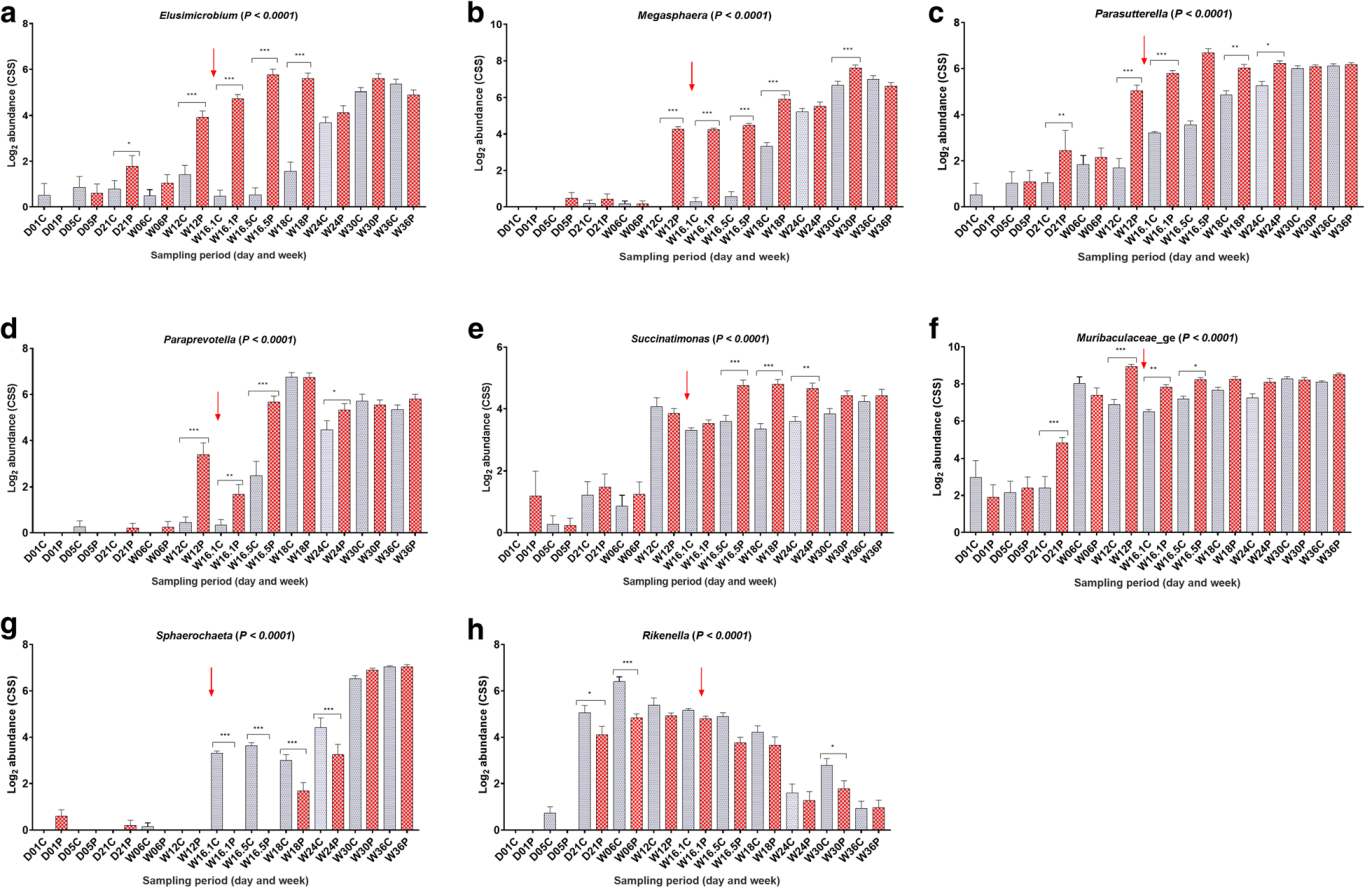
Abundance of gut microbiota at genus level affected by probiotic supplementation at multiple sampling periods. **a**). *Elusimicrobium*. **b**). *Megasphaera*. **c**). *Parasutterella*. **d**). *Paraprevotella*. **e**). *Succinatimonas*. **f**). *Sphaerochaeta*. **g**). *Muribaculaceae*_ge. **h**). *Rikenella*. In each panel of Fig. 4, the letters “D” and “W” refer to day and week post-hatch, while the letters “C” and “P” refer to control and probiotic supplemented flocks, respectively. Asterisks (*, ** and ***) show P values at 0.01, 0.001 and 0.0001, respectively

The overall abundance of microbial communities (genus level) significantly different between the probiotic supplemented and control flocks were further investigated to understand the effects of probiotic in a longitudinal manner. The data showed that the probiotic supplementation affected the composition of gut microbiota with significant effects observed from Day 21 until Week 30 of flock age at least for one or more microbial communities (Fig. 4). The abundance levels of *Elusimicrobium*, *Megasphaera*, *Parasutterella* and *Paraprevotella* (Fig. 4 a-d), and *Succinatimonas* and *Muribaculaceae*_ge (Fig. 4 e, f) were significantly higher in the probiotic flock at the respective sampling periods.

Probiotic supplementation also significantly reduced the abundance levels of *Sphaerochaeta* and *Rikenella* (Fig. 4 g, h). The data showed that *Sphaerochaeta* mainly colonised the gut around week 16, while *Rikenella* started appearing from Day 21 of chickens’ age. Overall, the age-wise analysis of the data showed that the colonisation time of different microbial communities varied with the nature of bacteria, where *Bacteroides*, *Rikenellaceae*_RC9_gut_group, *Lactobacillus*, *Alistipes*, *EscherichiaShigella*, *Enterococcus*, *Clostridium* sensu stricto and *Enterococcaceae*_unclassified were present in abundance in the gut from day one (Additional file 7: Fig. S3 and Additional file 8: Fig. S4). Interestingly, the abundance levels of *Bacteroides*, *Rikenellaceae*_RC9_gut_group, *Lactobacillus* and *Alistipes* (Additional file 7: Fig. S3 a – d) in the gut of both in the control and the probiotic flocks remained consistent until week 36, while *EscherichiaShigella*, *Enterococcus*, *Clostridium* sensu stricto and *Enterococcaceae*_unclassified decreased (Additional file 8: Fig. S4 a – d).

### Microbial community diversity affected by probiotic supplementation and flock age

#### Microbial alpha diversity

**Fig. 5:**
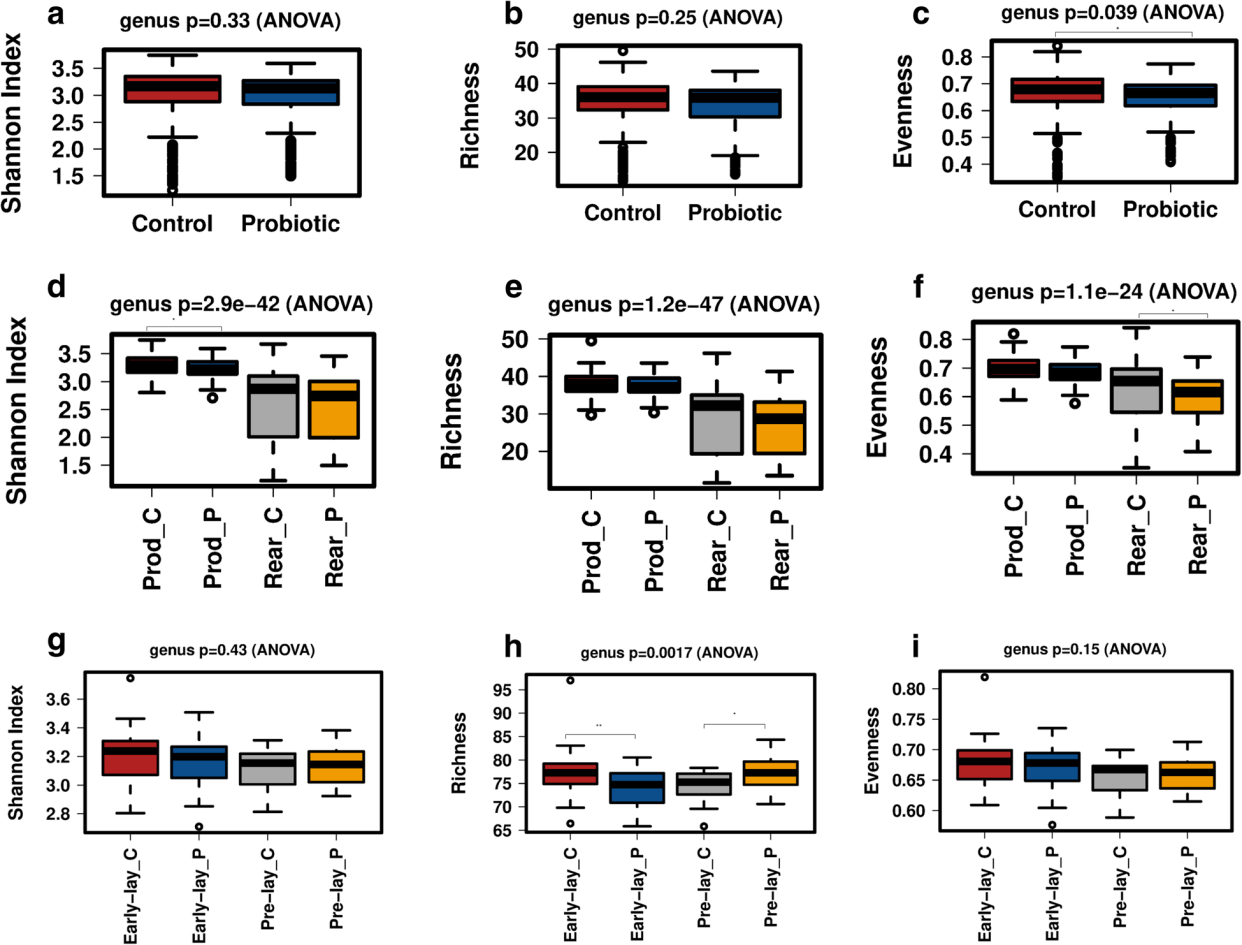
Alpha diversity of gut microbiota of laying chickens affected by probiotic supplementation, rearing and laying conditions. **a)**. Overall diversity of the probiotic and control flocks. **b)**. Overall richness of taxa of samples collected from control and probiotic supplemented flocks. **c)**. Overall evenness of taxa of samples collected from control and probiotic supplemented flocks. **d)**. Overall diversity affected by the probiotic supplementation in the rearing (Rear) and production (Prod) phases of laying chickens. **e)**. Overall richness of taxa of samples in Rear and Prod phases of laying chickens. **f)**. Overall evenness of taxa of samples in Rear and Prod phases of laying chickens. **g)**. Overall diversity affected by the probiotic supplementation in the Pre-lay and Early-lay phases of laying chickens. **h)**. Overall richness affected by the probiotic supplementation in the Pre-lay and Early-lay phases of laying chickens. **i)**. Overall evenness affected by the probiotic supplementation in the Pre-lay and Early-lay phases of laying chickens. Within each treatment group, “C” and “P” refer to control and probiotic supplemented flocks, respectively. Alpha diversity was measured at the genus level using Shannon index, richness and evenness in Calypso software. Asterisks (*) and (**) show a significant variation in community structure of samples collected from control and probiotic supplemented groups at *P* < 0.05 and *P* < 0.005, respectively

Shannon index is widely used to measure the alpha diversity of a community that includes both the number of present taxa (richness) and how evenly the taxa are distributed (evenness). Alpha diversity measures variation in the structure of microbial community within individual samples. Measured by the Shannon index and Richness, overall there was no significant variation (*P* > 0.05) in the community structure of individual samples collected from the probiotic and the control flocks (Fig. 5 a, b). However, taxa in the control group was significantly (*P* = 0.039) evenly distributed (Fig. 5 c). The community structure was affected both by the probiotic supplementation and rearing conditions as the diversity, richness and evenness were significantly different within the samples collected from flocks in rearing and production phases (Fig. 5 d - f). Hormonal changes occurring due to sexual maturity and on-set of lay might affect the diversity of gut microbiota. To understand this effect, the samples collected at week 12 to week 30 of flock age were categorised into pre-lay and early-lay periods and analysed for alpha diversity of gut microbiota. The Shannon index values showed that laying phase did not significantly affect the overall alpha diversity of the taxa of each treatment group (Fig. 5 g). However, the richness of the taxa in the control group before lay showed significant variation, while after the onset of lay, the variation was significantly higher in the samples collected from the probiotic supplemented group (Fig. 5 h). The evenness of the taxa was not significantly affected by the lay condition (Fig. 5 i).

**Fig. 6:**
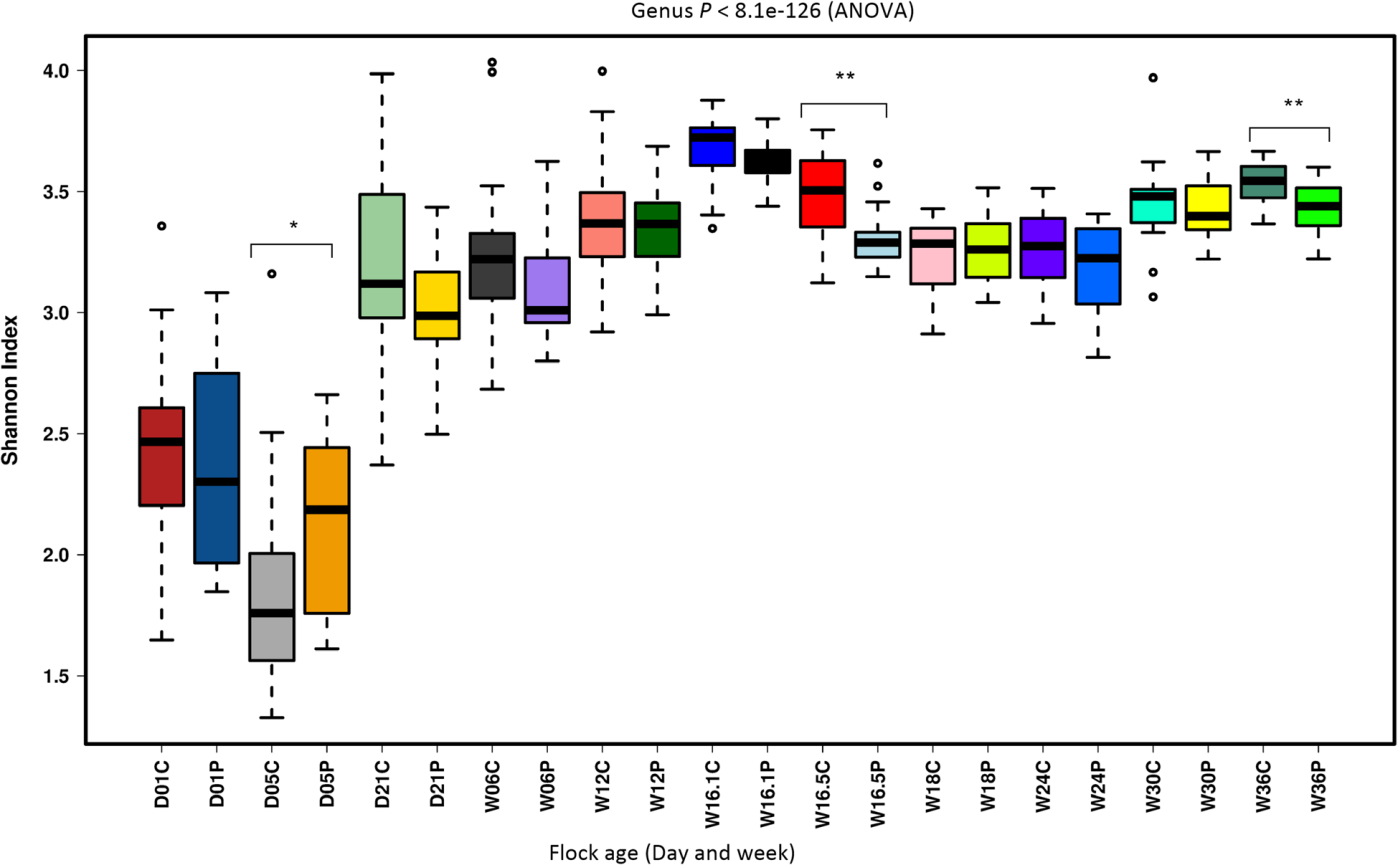
Alpha diversity of gut microbiota affected by probiotic supplementation and flock age. For direct comparison, alpha diversity at each sampling period was compared between the control and probiotic supplemented flocks. Letters “D” and “W” refer to flock age in a day and week post-hatch, while letters “C” and “P” refer to control and probiotic supplemented flock. Alpha diversity was measured at the genus level using the Shannon index in Calypso software that takes into account both the richness and evenness of microbial communities. Asterisks (* and **) show P values at 0.01 and 0.001, respectively. As per feed management guidelines of Hy-Line International, a different type of feed was introduced at 1, 7, 13, 15 and 18 weeks of flock age

The gut microbiota alpha diversity profile of the taxa of individual samples varied (*P* < 8.1e^− 126^) with flock age both in the probiotic supplemented and control flocks (Fig. 6). Both in the probiotic supplemented (*P* = 2.2e^− 99^) and control (*P* = 1.7e^− 78^) flocks, the alpha diversity of individual samples within each treatment group significantly varied from day 21 and onwards of flock age. Alpha diversity (measured by Shannon index) both in the control and probiotic supplemented groups increased from day 5 to week 16 before the flocks were moved to a free range farm for egg production. The probiotic supplemented flock showed a significantly lower alpha diversity on week 16.5 just after transportation to the production farm and on week 36 of flock age. This indicates that immediately after transportation, the alpha microbial diversity increased in the control group. For assessing the effects of feed on the alpha diversity of gut microbiota, the data were analysed on the basis of feed category phases, such as starter, grower, pre-lay and peak-lay. As expected, the overall alpha diversity (measured by Shannon index) showed significantly lower values (*P* = 7.2e^− 62^) in the starter phase of the feeding regimen (Additional file 9: Fig. S5 a). Richness (Additional file 9: Fig. S5 b) and evenness (Additional file 9: Fig. S5 c) also varied with feeding regimen and probiotic supplementation. As the birds were transported from rearing to the production farm, the samples obtained at week 12 and week 16 were assessed for the effects of stress on the alpha diversity of gut microbiota. Compared with the pre-transport, the post-transport individual samples diversity significantly varied (*P* = 0.0088) in the control flock (Additional file 9: Fig. S5 d). The richness of taxa at week 12 was significantly higher (*P* = 4.6e^− 07^) in the probiotic supplemented (Additional file 9: Fig. S5 e), while evenness was higher (*P* = 3.4e^− 06^) at week 16 in the control flock (Additional file 9: Fig. S5 f).

#### Microbial beta diversity

**Fig. 7:**
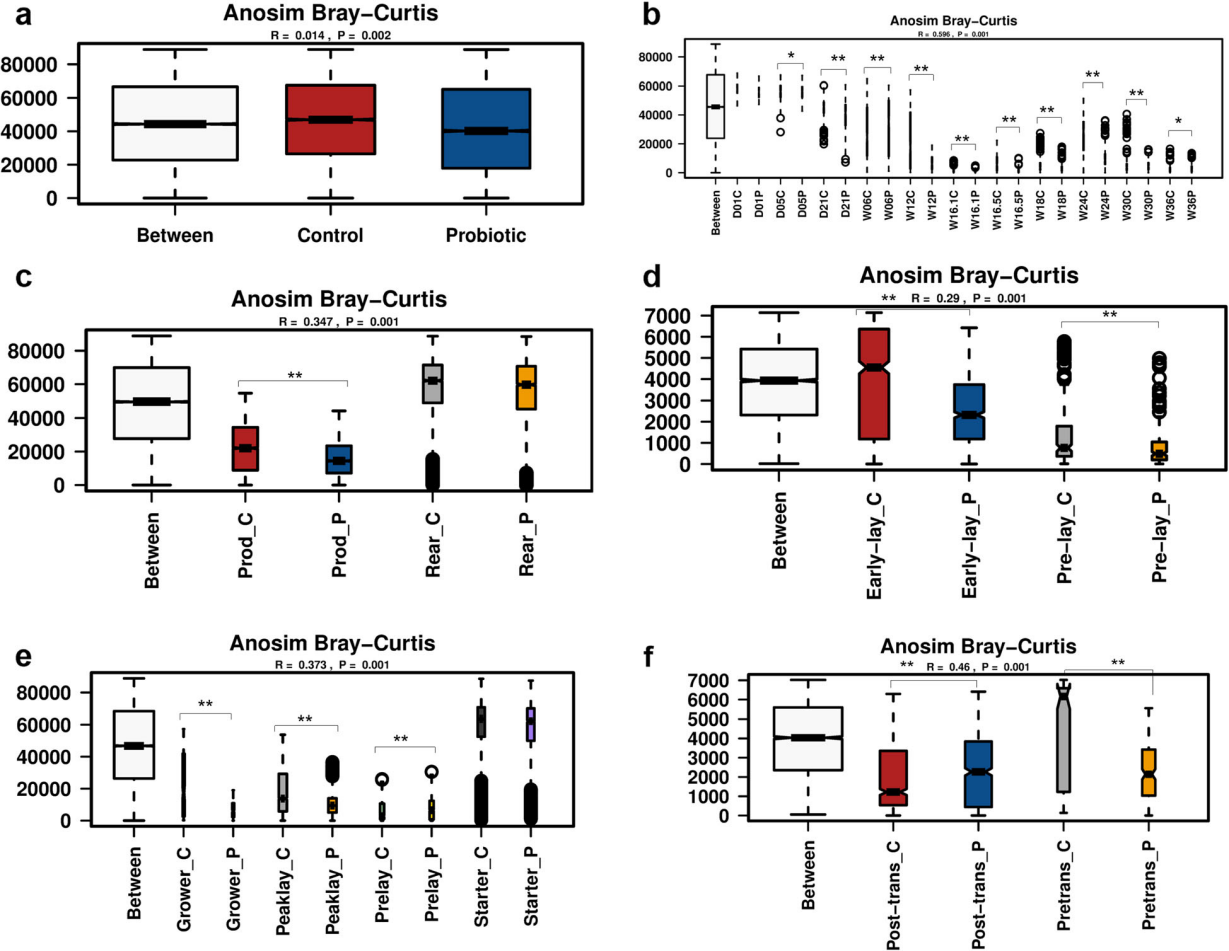
Beta diversity of gut microbiota of laying chickens affected by probiotic supplementation, flock age and rearing conditions. **a)**. Overall beta diversity of probiotic and control flocks. **b)**. Beta diversity affected by flock age. **c)**. Beta diversity affected by the probiotic supplementation in the rearing (Rear) and production (Prod) phases of laying chickens. **d)**. Beta diversity affected by the probiotic supplementation in the Pre-lay and Early-lay phases of laying chickens. **e)**. Beta diversity affected by the feeding regimen. **f)**. Beta diversity affected by transportation stress. Within each treatment group, “C” refers to control, while “P” refers to the probiotic supplemented flocks. Beta diversity was measured at genus level by ANOSIM Bray-Curtis dissimilarities matrix in Calypso software. Asterisks (**) show P value at 0.001 between the respective two treatment groups

Beta diversity is used to measure similarity or dissimilarity of microbial communities between samples and; therefore, is a useful technique to capture changes in community composition based on the ecosystem. Measured by the ANOSIM Bray-Curtis dissimilarities matrix, the beta diversity of the flock that received the probiotic was lower (*P* = 0.002) than the control flock (Fig. 7 a). Compared with the control, beta diversity was significantly lower in the probiotic supplemented group for the samples collected from day 5 on-wards (Fig. 7 b). Both in the control and probiotic supplemented groups, lowest diversity was recorded in the samples collected immediately post-transportation (16.1 week of flock age) to the production farm. Assessing the age effect on the beta diversity, the data showed that the community structure becomes less diverse with the flock age (Fig. 7 b). The beta diversity of the gut microbiota in the production was lower than the rearing phase, whereas the probiotic supplementation significantly reduced (*P* = 0.001) the beta diversity in the production phase of the chickens (Fig. 7 c). Comparing the beta diversity of the gut microbiota in the pre-lay and early-lay phases showed that probiotic supplementation reduced (*P* = 0.001) the beta diversity in both phases (Fig. 7 d). The feeding regimen data showed that the beta diversity of the gut microbiota was lower (*P* = 0.001) when the flocks were on prelay and peak lay diets (Fig. 7 e). However, these data should be interpreted carefully, as we did not sample the flocks on exact time-points just before and after changing the diets. Therefore, age might have been a confounding factor here. Interestingly, when the data were assessed for understanding the transportation stress effect, the probiotic supplemented flock showed lower (*P* = 0.001) diversity post-transportation (Fig. 7 f).

**Fig. 8:**
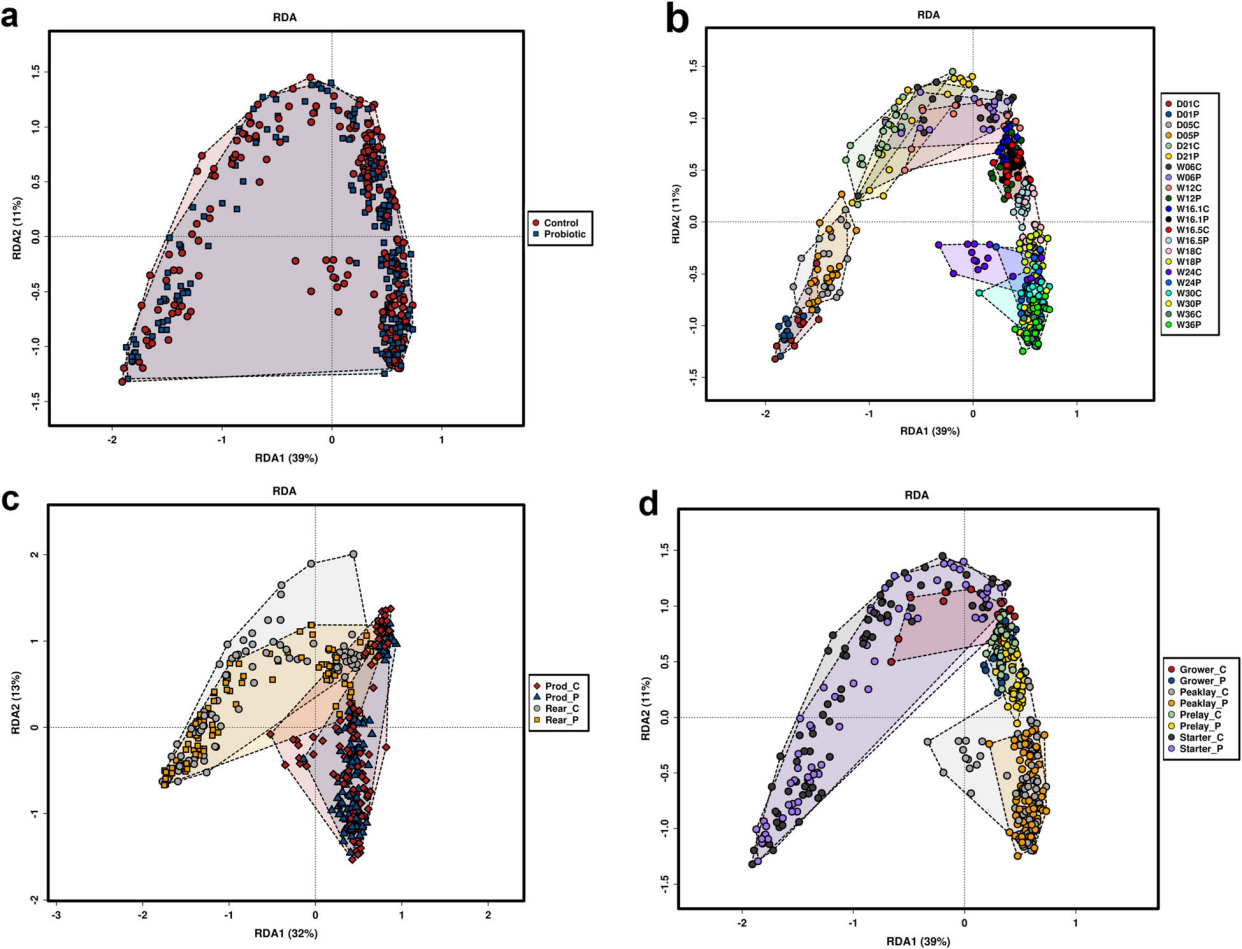
Genus level redundancy analysis showing gut microbiota composition affected by probiotic supplementation and flock arearing conditions. **a).** Overall community composition of gut microbiota between probiotic supplemented and control flocks. **b).** Community composition of gut microbiota affected by flock age and probiotic supplementation. **c).** Community composition of gut microbiota when the birds were sampled from a rearing (Rear) and production (Prod) farms. **d).** Community composition of gut microbiota affected by diet regimen. Within each treatment group, “C” refers to control, while “P” refers to the probiotic supplemented flocks. As per feed management guidelines of Hy-Line International, a different type of feed was introduced at week 1 (starter), week 7 (grower), week 13 (developer), week 15 (pre lay) and week 18 (peak lay) of flock age

To understand that how different was the microbial community structural composition between the probiotic and control groups collected at different time-points, redundancy analysis (RDA+) was performed. The community structure of the probiotic and control flocks overlapped with each other but was significantly (*P* = 0.005) different (Fig. 8 a). The community structure changed (*P* = 0.001) with flock age both in the probiotic supplemented and control flocks (Fig. 8 b), whereas rearing conditions had a profound effect (*P* = 0.001) on it (Fig. 8 c). The community structure changed (*P* = 0.001) in composition with a change in diet regimen (Fig. 8 d).

## Functional prediction of gut microbial communities based on *16S rRNA* data

**Fig. 9:**
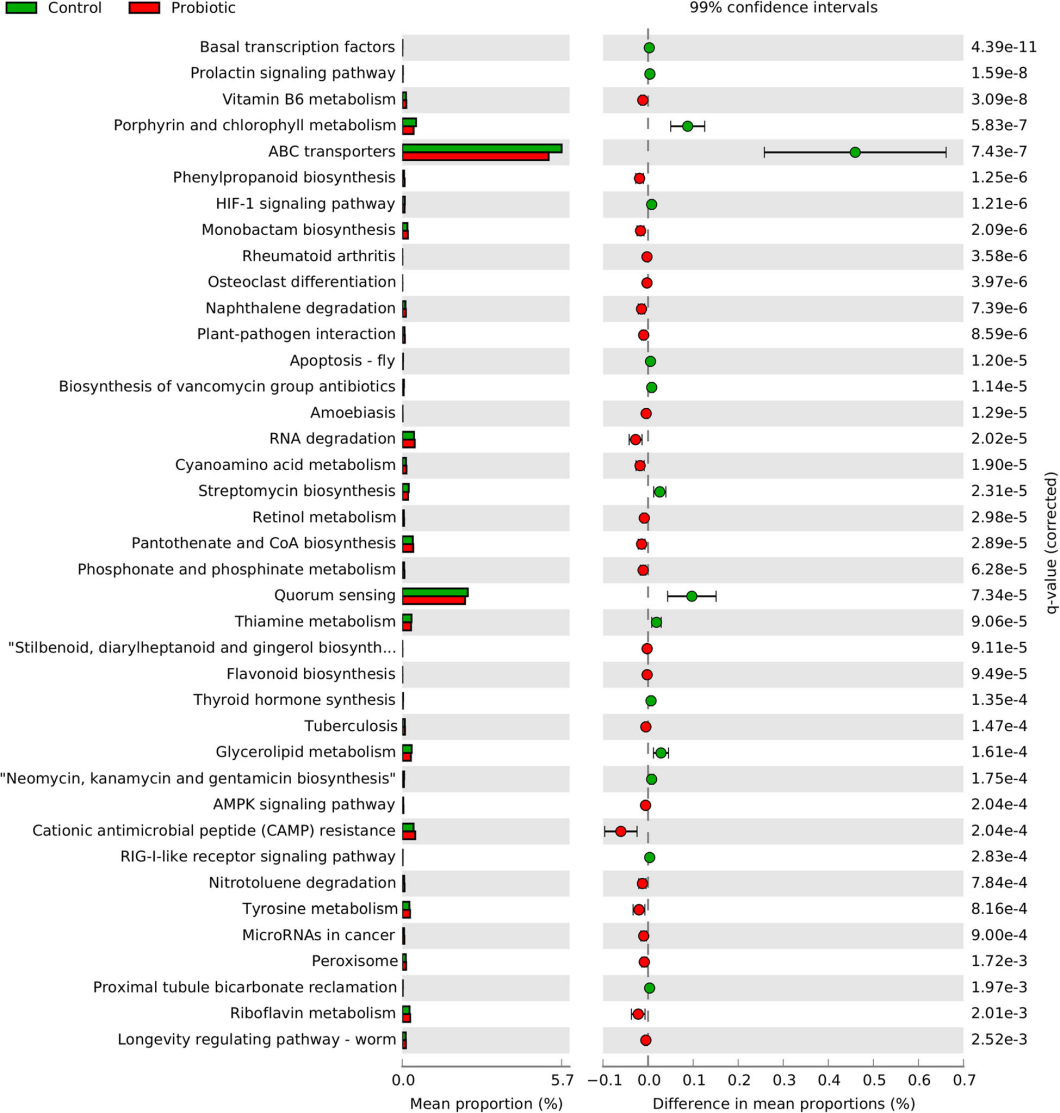
Metabolic pathways of the gut microbiota affected by probiotic treatment. To understand the effects of *Bacillus* based probiotic on the differential abundance of metabolic pathways of the gut microbiota, the functional prediction data obtained through Tax4Fun2 were analysed in STAMP by using Welch’s t test with 99% confidence interval. For multiple test correction in STAMP, Benjamini-Hochberg was used with a q-value filter > 0.05 that resulted only in the features that were significantly different between the two treatment groups. The remaining significantly enriched metabolic pathways belonging to Fig. 9 have been visualised in Additional file 11: Fig. S6

The effects of the probiotic on gut microbial metabolic pathways were assessed by performing functional predictions analysis in Tax4Fun2 using the KEGG orthologies (KOs) database and STAMP for Welch’s t-test for calculating the level of significance between the treatment groups. The data were mapped to 345 functional metabolic pathways (Additional file 10). Functional metabolic pathways, such as vitamin B6 metabolism, lipopolysaccharide biosynthesis, phenylpropanoid biosynthesis, monobactam biosynthesis, RNA degradation, retinol metabolism, pantothenate and CoA biosynthesis, phosphonate and phosphinate metabolism, AMPK signaling pathway, cationic antimicrobial peptide (CAMP) resistance and tyrosine metabolism were significantly enriched in the probiotic supplemented compared with the control flock (Fig. *9**).*

The functional prediction of metabolic pathways data were also compared between the probiotic supplemented and control flocks at rearing and production phases of life. Less number of significantly enriched pathways in the probiotic treatment group in the rearing was observed than in the production phase of the layer chickens (Additional file 12: Fig. S7 and Additional file 13: Fig. S8). The common metabolic pathways significantly enriched in the probiotic supplemented flock both in the rearing and production phases included retinol metabolism, pantothenate and CoA biosynthesis, 2-oxocarboxylic acid metabolism and naphthalene degradation. Metabolic pathways, such as tropane, piperidine and pyridine alkaloid biosynthesis, nitrogen metabolism, tyrosine metabolism, lipopolysaccharide biosynthesis, methane metabolism, nonribosomal peptide structures and C5-Branched dibasic acid metabolism were significantly enriched in the probiotic supplemented group in the rearing phase only (Additional file 12: Fig. S7). Metabolic pathways such as N-glycan biosynthesis, plant-pathogen interaction, RNA degradation, vitamin B6 metabolism, AMPK signaling pathway, “phenylalanine, tyrosine and tryptophan biosynthesis”, D-glutamine and D-glutamate metabolism, biosynthesis of amino acids, steroid degradation, biosynthesis of secondary metabolites and biosynthesis of antibiotics were only significantly enriched in the probiotic supplemented group at production phase of life (Additional file 13: Fig. S8).

## Flock performance affected by probiotic supplementation

The flock performance raw data obtained from the farm manager were visualised in Excel. The data showed that the weekly feed conversion ratio (FCR) was higher for the probiotic supplemented compared with the control flock (Additional file 14: Fig. S9). The egg production measured as the rate of lay (%) and egg weight (g) from week 18 until week 36 of flock age were not different between the control and probiotic supplemented flocks (Additional file 15: Fig. S10).

Average body weight (kg) measured on 100 birds at weekly intervals was not different between the control and probiotic supplemented flocks (Additional file 16: Fig. S11). Eggs collected at 24, 30 and 36 week of flock age were processed for egg quality parameters. The probiotic supplementation significantly (*P* < 0.05) improved egg internal quality but not shell quality (Additional file 17: Fig. S12 a – f). The overall quality of albumen height, Haugh Unit and yolk colour was significantly higher in the probiotic supplemented flock compared to the control flock (Additional file 17: Fig. S12 d – f).

## Effect of *Bacillus* based probiotic on *Salmonella* in free-range layer production

No *Salmonella* was isolated from the rearing and production sheds prior to placing the day-old chicks or point of lay pullets. Throughout the sampling period, *Salmonella* was isolated from one faecal sample and one environmental sample from the control shed at 18 and 36 weeks of flock age. At week 36, a shoe cover from the control shed was also positive for *Salmonella* spp. During the sampling period (day 1 to week 36 of flock age), no *Salmonella* was isolated from the probiotic supplemented shed. Measured through the mMPN method, a load of *Salmonella* in the faecal swab, environmental swab and shoe cover was 7.357, 11.170 and 15.06 per mL of BPW, respectively.

## *Salmonella* serotype confirmation through PCR

The PCR and agarose gel electrophoresis results confirmed that one faecal sample collected from the control shed at 18 weeks of flock age was *Salmonella* Typhimurium, while one each of the environmental and shoe cover samples collected at 36 weeks of flock age from the control shed was non Typhimurium serotypes (Additional file 18: Fig. S13).

## Discussion

In the laying hen industry, probiotics are mainly used as feed supplements for improving flock’s performance; therefore, there is a need to understand the effects of direct-fed microbials on the composition and development of gut microbiota both in the rearing and production phases of cage layers reared in the field conditions. Also, not many longitudinal field trials have investigated the effects of probiotics supplementations on the gut microbiota of free range hens. The composition and the development of gut microbiota rapidly change in the first few weeks of chicken age [17, 41, 42] and introducing the right type of probiotic candidate at hatch may influence it positively, which could reflect in better performance of the flock. Considering the continuous supplementation of *Bacillus* based probiotic for reduction of pathogen load in the faeces is important for flock productivity and food safety [6], in the current study, we investigated the role of *Bacillus* based probiotic in free range production system. The data showed that the probiotic was effective in the abundance of microbial genera of gut microbiota both in the rearing and production phases of layers. However, the gut microbiota composition also changed as the flocks get advanced in age.

At the phylum level, the composition of gut microbiota dominated by Bacteroidetes, Firmicutes and Proteobacteria show the important role of these bacteria in gut health. These data also show Proteobacteria is the third dominant phylum in the gut of chickens. In a previous study, Bacteroidetes, Firmicutes and Proteobacteria have been shown as the main dominant phyla in the caecal content of laying chickens [41]. A 3.07% increase in the overall population of Bacteroidetes in the probiotic supplemented flock shows its useful effects on gut health. Bacteroidetes are comprised of many bacteria that have the ability to digest complex substrates, such as xylan [43] and cellulose [44]. Microbial communities in gastrointestinal Bacteroidetes primarily produce propionate [45] and succinate [46] that are involved in intestinal gluconeogenesis. Proteobacteria was the third most abundant component of the gut microbiota with an overall 3.11% increase in the probiotic supplemented flock. This increase could be partly attributed to *Parasutterella*, which showed higher abundance in the probiotic flock. Proteobacteria also contain opportunistic pathogens, such as *Campylobacter*, *Escherichia*, *Shigella*, *Salmonella* and *Helicobacter*. In the current study, the abundance levels of these bacteria were more affected by flock age than the probiotic except *Helicobacter* that was significantly higher in the probiotic supplemented birds. The role of *Helicobacter* as a disease causing agent in chickens and its transmission through chicken meat and eggs needs to be investigated. A 5.95% decrease in the overall population of Firmicutes shows that the probiotic supplementation reduced the abundance of certain bacteria including Peptostreptococcus (0.323% in control versus 0.069% in probiotic). The increased abundance of Bacteroidetes in the gut at the production stage compared with the rearing both in the control and probiotic flocks shows age driven effects. Interestingly, the probiotic effects on the abundance level of Bacteroidetes were higher in the production compared with the rearing stage. The decreased abundance of Firmicutes after week 16 of flock age might explain the increase in Bacteroidetes that contains both succinate and propionate producing microbial communities.

The non-significant difference in alpha diversity (measured by Shannon index) between the probiotic supplemented and control flocks showed that microbial communities were more evenly distributed in the rearing phase. Probiotic supplementation did not significantly affect the alpha diversity (measured by Shannon index) of most of the faecal samples collected at different time-points. An overall significantly higher alpha diversity of microbial communities in production compared with the rearing phase highlights the role of rearing conditions. A steady increase in the alpha diversity index of the probiotic supplemented and control flocks with age shows that age had profound effect on community structure within chickens. However, beta diversity showed a reduction trend with flock age, suggesting that as the chickens increased in age, the variation in the gut microbial communities between birds reduced. Beta diversity was also lower for the probiotic supplemented flock (Anosim Bray-Curtis *P* = 0.001). Interestingly, assessing the effects of transportation of birds from rearing to production sheds on the beta diversity of gut microbiota showed that variation in the community structure increased in the probiotic supplemented flock post-transportation.

The intestinal microbiota is involved in the fermentation process of complex carbohydrates, such as indigestible dietary fibers and sugars to generate SCFAs that help in maintaining a healthy gut environment [13]. In the current study, overall, probiotic supplementation increased the abundance levels of many microbial communities in the gut. The increased abundance levels of *Parasutterella*, *Paraprevotella*, *Megasphaera*, *Elusimicrobium*, *Succinatimonas*, *Desulfovibrionaceae*_unclassified and *Muribaculaceae*_ge show that chickens performance was enhanced as these bacteria are involved in a range of useful functions in the gut of the host. Characterised in the gut of mice, *Parasutterella* plays a role in the maintenance of bile acid and cholesterol metabolism [47]. In our previous study, probiotic supplementation increased the abundance level of *Parasutterella* in the gut of *Salmonella* Typhimurium challenged laying chickens [6]. *Parasutterella* has shown to increase in abundance in the gut of pigs that received a prebiotic (waxy corn starch) supplemented diet [48], and in the gut of SPF chickens that received *Lactobacillus casei* supplemented diet [49]. In laying chickens gut, *Paraprevotella* is involved in polysaccharide degradation, and propionate and butyrate production through the expression of xylose isomerase, cobalamin-binding methylmalonyl-CoA mutase and/or methylmalonyl-CoA epimerase and acetyl-CoA acetyltransferase, respectively [50]. *Megasphaera* preferably produces butyrate through the production of acetyl-CoA acetyltransferase [50]. *Elusimicrobium* is a member of the phylum Elusimicrobia, which was the least abundant both in the control and probiotic supplemented flocks. *Elusimicrobium minutum* is the only identified specie in the Elusimicrobia and is an obligatory anaerobic ultramicrobacterium that ferments glucose [51]. *Succinatimonas* is a strict anaerobe that produces succinate and acetate from carbohydrates [52]. Members of Desulfovibrionaceae oxidise organic substrates incompletely to acetate [53]. In 60-week old Hy-Line Brown layers, the probiotic *B. licheniformis* alleviated the adverse influence of heat on egg production and gut health [54]. *Bacillus subtilis* strain DSM29784 has been shown to selectively increase the abundance of gut microbial genera, such as *Bifidobacterium, Lactobacillus* and *Alistipes* in different growing stages of layers [55]. The current study shows that the increased abundance of many of these microbial genera in the gut shows the useful aspects of the *Bacillus* based probiotic supplementation in the diet of layers.

In the functional prediction data, the significant enrichment of metabolic pathways, such as vitamin B6 metabolism, cyanoamino acid metabolism, retinol metabolism, lipopolysaccharide metabolism and biosynthesis of secondary metabolites show that probiotic supplementation improved gut functions. Vitamin B6 contributes to intestinal immune regulation through the metabolism of lipid mediator sphingosine 1-phosphate. Microbial vitamin B6 is synthesized as pyridoxal phosphate from d-ribulose 5-phosphate and glyceraldehyde-3-phosphate or 4-phosphohydroxy-L-threonine and deoxyxylulose 5-phosphate [56]. Several natural isolates and engineered bacteria including Bacillus subtilis, produce vitamin B6 [57].

Different strains of probiotics vary in their mechanistic actions; therefore, we have only compared the findings of the current study with studies that used *Bacillus* based probiotics in layers. In laying hens, diet supplemented with *Bacillus* based probiotics has shown to improve egg production and overall egg quality. For example, supplementation of *Bacillus subtilis* but not *Bacillus licheniformis* in layer diet resulted in improved egg internal quality and egg production [58]. Feeding *Bacillus licheniformis* to a 28-week old layers flock for up to 8 weeks resulted in increased egg production, shell thickness and better intestinal epithelial cell morphology [59]. In 24-wk-old Lohmann Pink layers, *Bacillus subtilis* supplementation for up to 8 weeks resulted in improved FCR and reduced serum cholesterol level [60]. In the current study, the significantly higher albumen height, Haugh Unit and yolk colour show the positive effects of the *Bacillus* based probiotic on egg internal quality parameters; however, it is worth mentioning that the egg quality was measured only at week 24, 30 and 36 weeks of flock age. The positive effects of probiotics could be due to improved nutrient absorption in the gut [61, 62] or through the production of metabolites, enzymes or vitamins [63, 64]. Egg quality deteriorates with flock age; therefore, future research can focus on the use of probiotics for improving gut health and birds performance from hatch to the end of the production cycle.

No *Salmonella* detected prior to the placement of day-old chicks on rearing and 16-week pullets on production farms demonstrates that the decontamination procedures performed were appropriate. Effective cleaning and disinfection of poultry sheds reduce the levels of *Salmonella* contamination; however, the recovery of *Salmonella* spp. from surfaces such as dropping boards and floors in cleaned and disinfected sheds is variable [65]. In a previous study, different *Salmonella* serotypes such as, *Salmonella* Mbandaka and *Salmonella* Typhimurium were isolated from a free-range layer production system [65]. In the current study, no *Salmonella* isolation from the probiotic supplemented shed could be attributed to the beneficial effects of probiotics; however, a conclusive statement could not be made as the flock was not followed beyond 36 week of age. Moreover, the number of *Salmonella* positive samples were low (*n* = 3); therefore, it is hard to estimate the true effects of the *Bacillus* based probiotic on the flock *Salmonella* spp. status. In a pen trial, feeding of *Bacillus* based probiotic reduced the overall load of *Salmonella* in faeces but not all of the birds turned negative for *Salmonella* in the 12 week period of sampling [6].

## Conclusions

In this field study, the most abundant bacterial phyla were Bacteroidetes, Firmicutes, and Proteobacteria in the gut microbiota. The probiotic supplementation from day-old significantly decreased overall abundance levels (%) of Firmicutes and Spirochaetes and increased Elusimicrobia. At the genus level, the higher abundance of Elusimicrobium, Megasphaera, Parasutterella, Desulfovibrionaceae_unclassified, Paraprevotella, Succinatimonas, bacteria_unclassified, and Muribaculaceae_ge in the probiotic supplemented flock shows its positive effects on gut health. The functional enrichment of metabolic pathways including cationic antimicrobial peptide (CAMP) resistance, vitamin B6 metabolism, AMPK signaling pathway, monobactam biosynthesis, RNA degradation, and tyrosine metabolism highlights the positive effects of the *Bacillus* based probiotic on the gut. The improvement in egg internal quality parameters suggests that the *Bacillus* based probiotic could be used continuously in a cage-free poultry production system. Exploring suitable alternative multidisciplinary programs that encompass efficient management as well as optimize nutrition and disease management is of great interest to the poultry industry. Findings from this study can play a significant role in creating an antibiotic-free production environment.

## Supplementary Information

**Additional file 1.** Layer chicken diet composition, isolation of *Salmonella* from swabs, faecal DNA extraction and traditional PCR.

**Additional file 2.** Quality of reads generated in the study.

**Additional file 3 Fig. S1.** Rarefaction curve analysis of OTUs depicting the quality of the *16S rRNA* reads generated from DNA obtained from laying chickens faeces. **a)** Rarefaction curve analysis of the reads based on the probiotic supplemented and control cohorts. **b)** Rarefaction curve analysis of the reads based on samples collected at Day (D) and Week (W) post-hatch from the probiotic supplemented (P) and control (C) groups. The flatten curves towards right show that the underlying microbial communities were well covered by the sequenced data.

**Additional file 4.** OTU Table containing details of taxa of faecal samples collected from the probiotic supplemented and control groups at different sampling time-points. (SUMMARY 1369 kb)

**Additional file 5.** Metadata file required for the OTU Table for further analysis and data visualisation. (CSV 52 kb)

**Additional file 6 Fig. S2.** Overall gut microbiota composition at phylum level in both the probiotic supplemented and control flocks pooled together. Microbial abundance (%) in the probiotic supplemented group. For percent calculation of microbial abundance at phylum level, total sum scaling normalised but untransformed data obtained from Calypso software were visualised in Excel 2016 and panel graphs were prepared in Graphpad prism v. 8.0.0.

**Additional file 7 Fig. S3.** Abundance of gut microbiota at genus level affected by probiotic supplementation at multiple sampling periods. a). *Bacteroides*. b). *Rikenellaceae*_RC9_gut_group. c). *Lactobacillus*. d). *Alistipes*. In each panel of the Supplementary Fig. S3, the letters “D” and “W” refer to day and week post-hatch, while the letters “C” and “P” refer to control and probiotic supplemented groups, respectively.

**Additional file 8 Fig. S4.** Abundance of gut microbiota at genus level affected by probiotic supplementation at multiple sampling periods. a). *EscherichiaShigella*. b). *Enterococcus*. c). *Clostridium* sensu stricto. d). *Enterococcaceae*_unclassified. In each panel of the Supplementary Fig. S4, the letters “D” and “W” refer to day and week post-hatch, while the letters “C” and “P” refer to control and probiotic supplemented groups, respectively.

**Additional file 9 Fig. S5.** Alpha diversity of gut microbiota of laying chickens affected by probiotic supplementation, rearing and laying conditions. **a)**. Alpha diversity (Shannon index) of gut microbiota affected by feeding regimen. **b)**. Richness of gut microbiota affected by feeding regimen. **c)**. Evenness of gut microbiota affected by feeding regimen. **d)**. Alpha diversity (Shannon index) of gut microbiota affected by transport stress. **e)**. Richness of gut microbiota affected by transport stress. **f)**. Evenness of gut microbiota affected by transport stress. Within each treatment group, “C” refers to control, while “P” refers to the probiotic supplemented cohort. Alpha diversity was measured at genus level by Shannon index, Richness and Evenness in Calypso software. Within each treatment group at specific sampling period, asterisk (*) shows a significant difference at *P* < 0.01, while asterisks (**) show *P* < 0.001.

**Additional file 10.** Details of metabolic pathways included in the functional prediction analysis. (XLS 3419 kb)

**Additional file 11 Fig. S6.** Metabolic pathways of the gut microbiota affected by probiotic supplementation. To understand the effect of *Bacillus* based probiotic on the differential abundance of metabolic pathways of the gut microbiota, the functional prediction data obtained through Tax4Fun2 were analysed in STAMP by using Welch’s t test with 99% confidence interval. For multiple test correction in STAMP, Benjamini-Hochberg was used with q value filter > 0.05 that resulted only in the features that were significantly different between the two treatment groups.

**Additional file 12 Fig. S7.** Metabolic pathways of the gut microbiota affected by probiotic supplementation in the rearing phase of laying flock. To understand the effect of *Bacillus* based probiotic on the differential abundance of metabolic pathways of the gut microbiota, the functional prediction data obtained through Tax4Fun2 were analysed in STAMP by using Welch’s t test with 99% confidence interval. For multiple test correction in STAMP, Benjamini-Hochberg was used with q value filter > 0.05 that resulted only in the features that were significantly different between the two treatment groups. In the figure, titles Rear_C and Rear_P refer to faecal samples collected from the control and probiotic supplemented cohorts at rearing phase of flock age, respectively.

**Additional file 13 Fig. S8.** Metabolic pathways of the gut microbiota affected by probiotic supplementation in the rearing phase of laying flock. To understand the effect of Bacillus based probiotic on the differential abundance of metabolic pathways of the gut microbiota, the functional prediction data obtained through Tax4Fun2 were analysed in STAMP by using Welch’s t test with 99% confidence interval. For multiple test correction in STAMP, Benjamini-Hochberg was used with q value filter > 0.05 that resulted only in the features that were significantly different between the two treatment groups. In the figure, titles Prod_C and Prod_P refer to faecal samples collected from the control and probiotic supplemented cohorts at production phase of flock age, respectively.

**Additional file 14 Fig. S9.** Feed conversion ratio of the probiotic supplemented and control flocks. Weekly (W) feed conversion ratio was calculated from feed intake and flock body weight.

**Additional file 15 Fig. S10.** Lay rate and egg weight of probiotic supplemented and control flocks.

**Additional file 16 Fig. S11.** Body weight of probiotic supplemented and control flocks. Body weight of 100 randomly selected chickens was taken weekly (W) from each of the probiotic supplemented and control flocks.

**Additional file 17 Fig. S12.** Egg quality parameters of eggs collected from probiotic supplemented and control cohorts. **a)** Egg weight (g). **b)** Shell weight (g). **c)** Shell thickness (mm). **d)** Albumen height (mm). **e)** Haugh Unit. **f)** Yolk colour. Freshly laid eggs were collected at week 24, 30 and 36 week of flock age and immediately analysed for egg shell and internal quality parameters.

**Additional file 18 Fig. S13. PCR products of** ***Salmonella*** **positive samples visualised on 2% agarose gel. L)** Ladder; **1)** *Salmonella* Typhimurium isolated from faecal swab at week 18 of flock age from the control shed. **2)** *Salmonella* spp. isolated from environmental swab isolated at week 36 of flock age from the control shed; **3)** *Salmonella* spp. isolated from environmental swab isolated at week 36 of flock age from the control shed. **4)** *Salmonella* Typhimurium as positive control. For *Salmonella* spp. typing primer pair (605 bp) amplifying the fragment of *invA* gene were used, while for *Salmonella* Typhimurium confirmation, primer pair (303 bp) amplifying the fragment of *TSR3* gene were used.

## Data Availability

The 16S rRNA sequence data are available from the NCBI SRA under the BioProject accession number PRJNA647475. The sample metadata and OTU Tables are provided as Supplementary data files 1–2 for reproducibility of this study.

## Abbreviations

BPW: Buffered peptone water
CSS: Cumulative-sum scaling
KEGG: Kyoto Encyclopedia of genes and genomes
NCBI: National Centre for Biotechnology Information
OTU: Operational taxonomic unit
RVS: Rappaport-Vassiliadis soya peptone
SCFAs: Short chain fatty acids
SRA: Sequence read archive
STAMP: Statistical analysis of taxonomic and functional profile
