# Supplementary material for: Functional enrichment of gut microbiome by early supplementation of Bacillus based probiotic in cage free hens: a field study
Source: Anim Microbiome. 2021 Jul 27;3:50. doi: 10.1186/s42523-021-00112-5 (PMC8314476; doi:10.1186/s42523-021-00112-5)
Supplement: Supplementary file 1 — Additional file 1. Layer chicken diet composition, isolation of Salmonella from swabs, faecal DNA extraction and traditional PCR. [file 42523_2021_112_MOESM1_ESM.docx]

**Supplementary file 1.**

**Processing of faecal and shed environmental samples for *Salmonella***

At least 0.5 mL from the individual faecal and environmental swab samples were stored at 5°C for the quantitative assessment of *Salmonella* by using the miniaturised most probable number (MPN) method. MPN was performed only on *Salmonella* confirmed positive samples. For *Salmonella* detection, an enrichment method previously described was followed [1, 2]. The enrichment process included an overnight incubation at 37°C of the swab samples collected in BPW, then 0.1 mL of the incubated BPW enrichment into 10 mL Rappaport Vassiliadis Soya Peptone Broth (RVS; ThermoFisher Scientific, Australia) and overnight incubation at 42°C for the selective growth of *Salmonella*. The incubated RVS samples were streaked into both xylose lysine deoxycholate (XLD; ThermoFisher Scientific, Australia) and brilliance *Salmonella* (BS) agar and incubated overnight at 37°C. The plates were read for the presence of *Salmonella* characteristic colony colour and morphology. A single colony from the *Salmonella* positive plates was sub-cultured in Lauria Bertani (LB; ThermoFisher Scientific, Australia) broth and stored in 50% glycerol for serotype determination through traditional PCR.

**PCR characterization of *Salmonella* serotypes**

The stored isolates were revived on nutrient agar (NA; ThermoFisher Scientific, Australia) and a single colony of *Salmonella* from the agar plates was grown in LB broth at 37°C in a shaking incubator. To obtain the bacterial pellet, the culture samples were centrifuged at 10000 ×*g* for 5 min, the supernatant discarded and the pellet was re-suspended in 0.2 mL of 6 % Chelex (Biorad, Australia) prepared in Tris–EDTA (TE) buffer. The samples were incubated at 56°C for 20 min, vortexed and incubated again at 95°C for 8 minutes. The samples were incubated on ice for 5 minutes, centrifuged briefly and the supernatant that containing DNA was stored at −20 °C until used for traditional PCR. A duplex PCR was performed to identify the serotype of the isolates collected during the sampling. Isolates were confirmed as *Salmonella* through the amplification of an *invA* gene fragment (Forward: 5′-AAACCTAAAACCAGCAAAGG-3′; Reverse: 5′-TGTACCGTGGCATGTCTGAG-3′). To confirm for *Salmonella* Typhimurium serotype, primers designed from the *TSR3* gene (Forward: 5′-TTTACCTCAATGGCGGAACC-3′; Reverse: 5′-CCCAAAAGCTGGGTTAGCAA-3′) were used in the same reaction well. PCR reactions were performed in a total volume of 25 µL that contained 5 µL of 5× MyRed Taq Buffer (Bioline, Australia), 0.3 μL of MyRed Taq polymerase, 1.25 µL of each of the forward and reverse primers for *invA*, 0.625 µL of each of the forward and reverse primers for *TSR3*, 13.95 μL PCR grade water and 2 µL of DNA template. PCR cycling conditions were: initial denaturation at 94°C for 2 minutes, then 40 cycles of 95°C for 30 s and 60°C for 30 s, followed by an extension of 72 °C for 5 min at the end. The PCR products were visualised on 2 % agarose gel electrophoresis to confirm the size and specificity of the bands.

**Enumeration of *Salmonella* through MPN**

The micro-dilution tube MPN method previously described [3] was used to enumerate *Salmonella* in positive samples. Briefly, 0.1 mL of the BPW samples were serially diluted (10^-1^ to 10^-8^) in 0.9 mL PBS, and 0.1 mL of each dilution was added (in triplicate) to the micro-dilution tubes containing 0.9 mL semi-solid RVS medium with the MRSV *Salmonella* selective agent (ThermoFisher Scientific, Australia) and incubated overnight at 42°C. White colour development indicated presumptive positive *Salmonella* growth, which was further confirmed on BS agar. A combination of positive and negative micro-dilution tubes gave the MPN result. MPN/mL was determined using the MPN tables sourced from the FDA Laboratory Methods[4].

**Quality DNA extraction from chicken faeces**

The recommended protocol of the QIAamp FAST DNA Mini Kit (Qiagen, Australia) did not yield pure and concentrated DNA; therefore, an optimised protocol was developed during a pilot trial. Briefly, approx. 200 mg individual faecal samples were mixed with 0.7 mL InhibitEx Buffer and vortexed for 1 min. One scoup each of acid-washed ≤ 106 μm and 425-600 μm (Sigma Aldrich, Australia) glass beads was added into each sample and the samples were homogenised for 5 min at speed 3 in bullet blender (Next Advances, USA). The samples were heated for 7 min at 80°C and centrifuged at 18000 ×*g* for 1.5 min to pellet down undissolved materials. The maximum supernatants were transferred into 1.5 mL tubes and centrifuged at 18000 ×g for 1.5 min to pellet down any remaining undissolved faecal materials. From the supernatant, 450 μL was added into 1.5 mL tubes containing 30 μL proteinase K (20 mg/ mL), vortexed briefly into which 400 μL Buffer AL was added. The samples were incubated at 70°C for 10 min with intermittent mixing and briefly centrifuged. Into each sample, 400 μL of ethanol (96–100%) was added, mixed by inversion and passed through the spin columns. DNA in the spin columns was washed with 500 μL of each of wash buffers AW1 and AW2 as per protocol of the kit. DNA was eluted in 100 μL Buffer AE (pre-heated at 55-60°C for 1 min) as per protocol of the kit. The average 260/280 and 260/230 ratios of the DNA in nanodrop-1000 were 1.9 and 2.3, respectively and the concentration was between 50 and 1200 ng/μL. The DNA concentration for the muconium samples was approximately 10 ng/μL.

**References**

1. Gole VC, Woodhouse R, Caraguel C, Moyle T, Rault J-L, Sexton M, Chousalkar K: **Dynamics of Salmonella shedding and welfare of hens in free-range egg production systems**. *Appl Environ Microbiol* 2017, **83**(5).

2. Khan S, Chousalkar KK: **Salmonella Typhimurium infection disrupts but continuous feeding of Bacillus based probiotic restores gut microbiota in infected hens**. *J Anim Sci Biotechnol* 2020, **11**(1):1-16.

3. Pavic A, Groves P, Bailey G, Cox J: **A validated miniaturized MPN method, based on ISO 6579: 2002, for the enumeration of Salmonella from poultry matrices**. *J Appl Microbiol* 2010, **109**(1):25-34.

4. Blodgett R: **BAM Appendix 2: most probable number from serial dilutions, Bacteriological Analytical Manual**. *Food and Drug Administration, Silver Spring, MD* [*https://www*](https://www) *fda gov/food/foodscienceresearch/laboratorymethods/ucm109656 htm* 2010.
