# Supplementary figures and images for: Functional enrichment of gut microbiome by early supplementation of Bacillus based probiotic in cage free hens: a field study
Source: Anim Microbiome. 2021 Jul 27;3:50. doi: 10.1186/s42523-021-00112-5 (PMC8314476; doi:10.1186/s42523-021-00112-5)

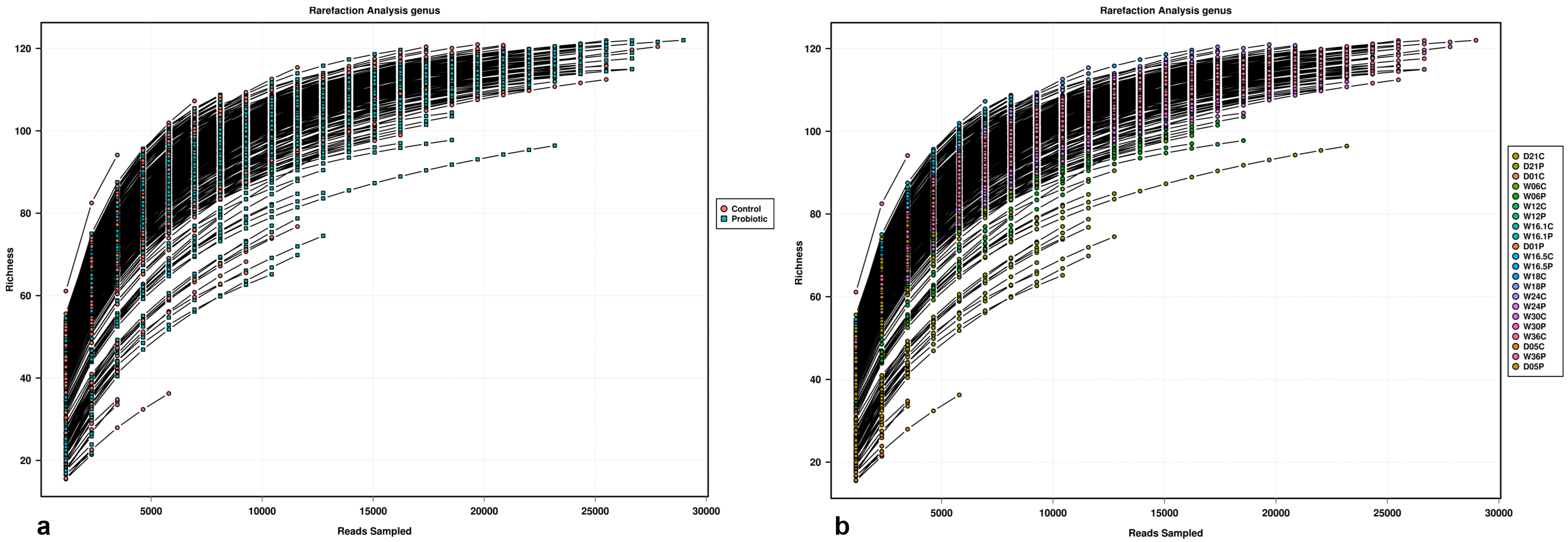

Supplement: Supplementary file 3 — Additional file 3 Fig. S1. Rarefaction curve analysis of OTUs depicting the quality of the 16S rRNA reads generated from DNA obtained from laying chickens faeces. a) Rarefaction curve analysis of the reads based on the probiotic supplemented and control cohorts. b) Rarefaction curve analysis of the reads based on samples collected at Day (D) and Week (W) post-hatch from the probiotic supplemented (P) and control (C) groups. The flatten curves towards right show that the underlying microbial communities were well covered by the sequenced data. [file 42523_2021_112_MOESM3_ESM.tif]

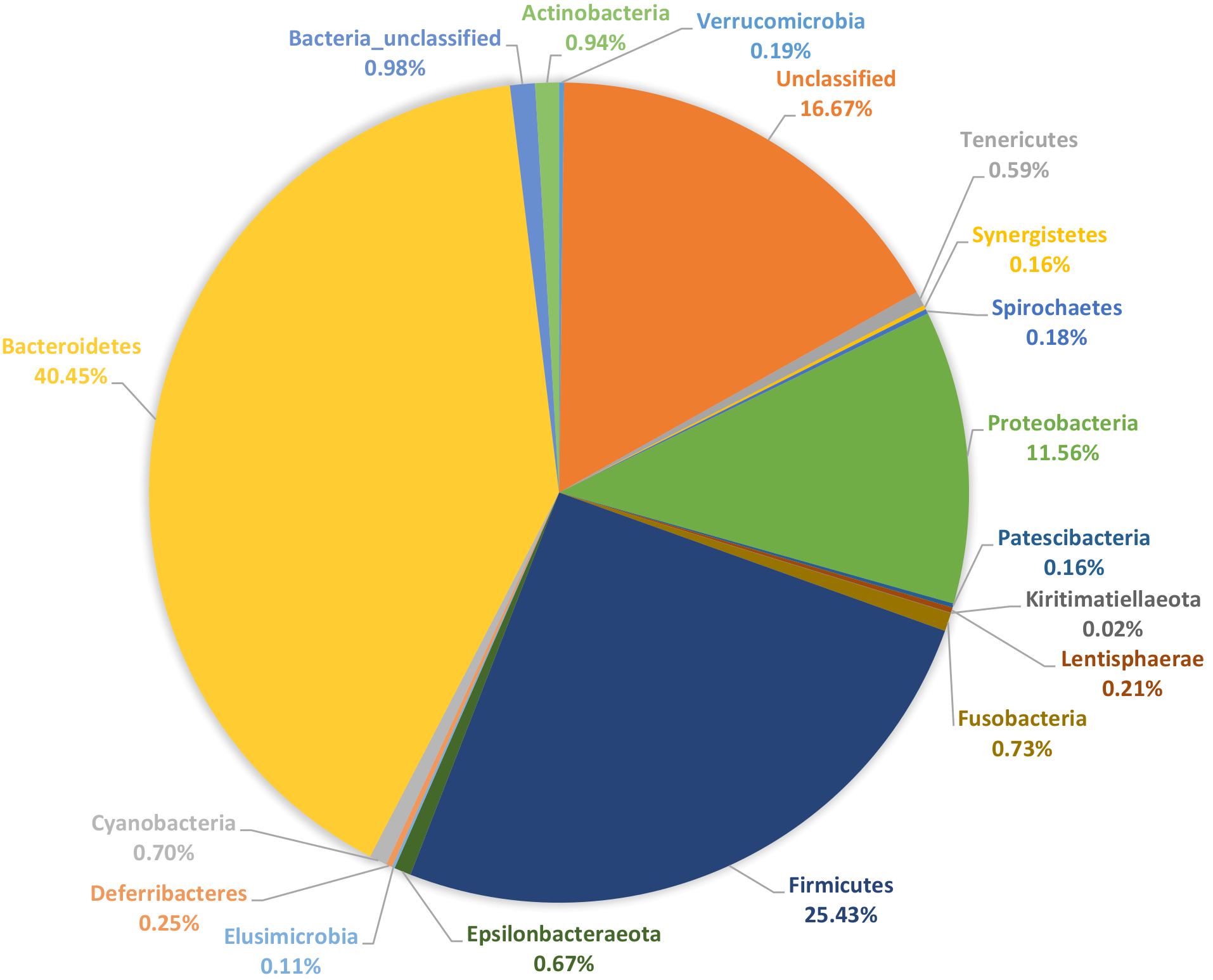

Supplement: Supplementary file 6 — Additional file 6 Fig. S2. Overall gut microbiota composition at phylum level in both the probiotic supplemented and control flocks pooled together. Microbial abundance (%) in the probiotic supplemented group. For percent calculation of microbial abundance at phylum level, total sum scaling normalised but untransformed data obtained from Calypso software were visualised in Excel 2016 and panel graphs were prepared in Graphpad prism v. 8.0.0. [file 42523_2021_112_MOESM6_ESM.tif]

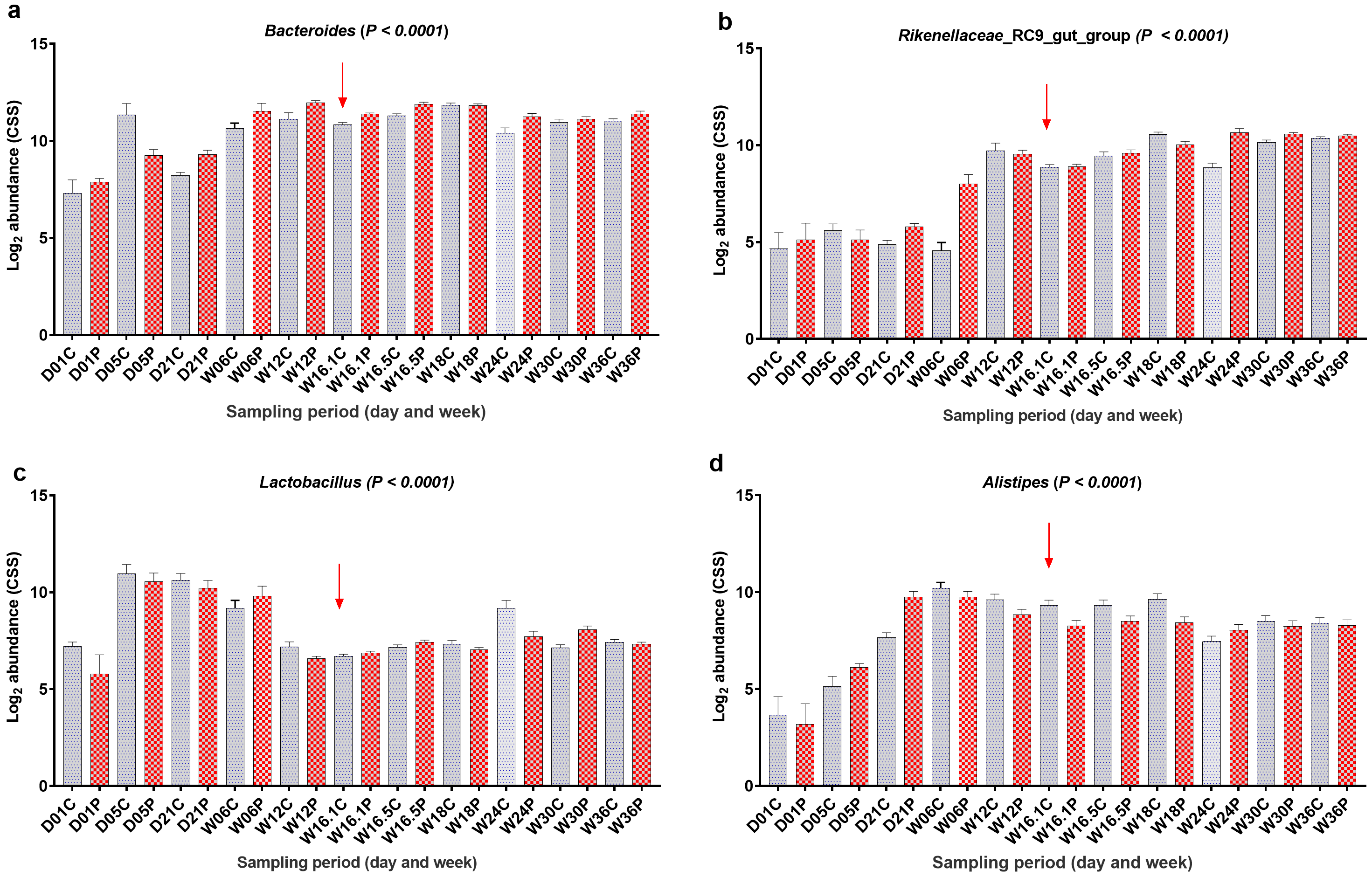

Supplement: Supplementary file 7 — Additional file 7 Fig. S3. Abundance of gut microbiota at genus level affected by probiotic supplementation at multiple sampling periods. a). Bacteroides. b). Rikenellaceae_RC9_gut_group. c). Lactobacillus. d). Alistipes. In each panel of the Supplementary Fig. S3, the letters “D” and “W” refer to day and week post-hatch, while the letters “C” and “P” refer to control and probiotic supplemented groups, respectively. [file 42523_2021_112_MOESM7_ESM.tif]

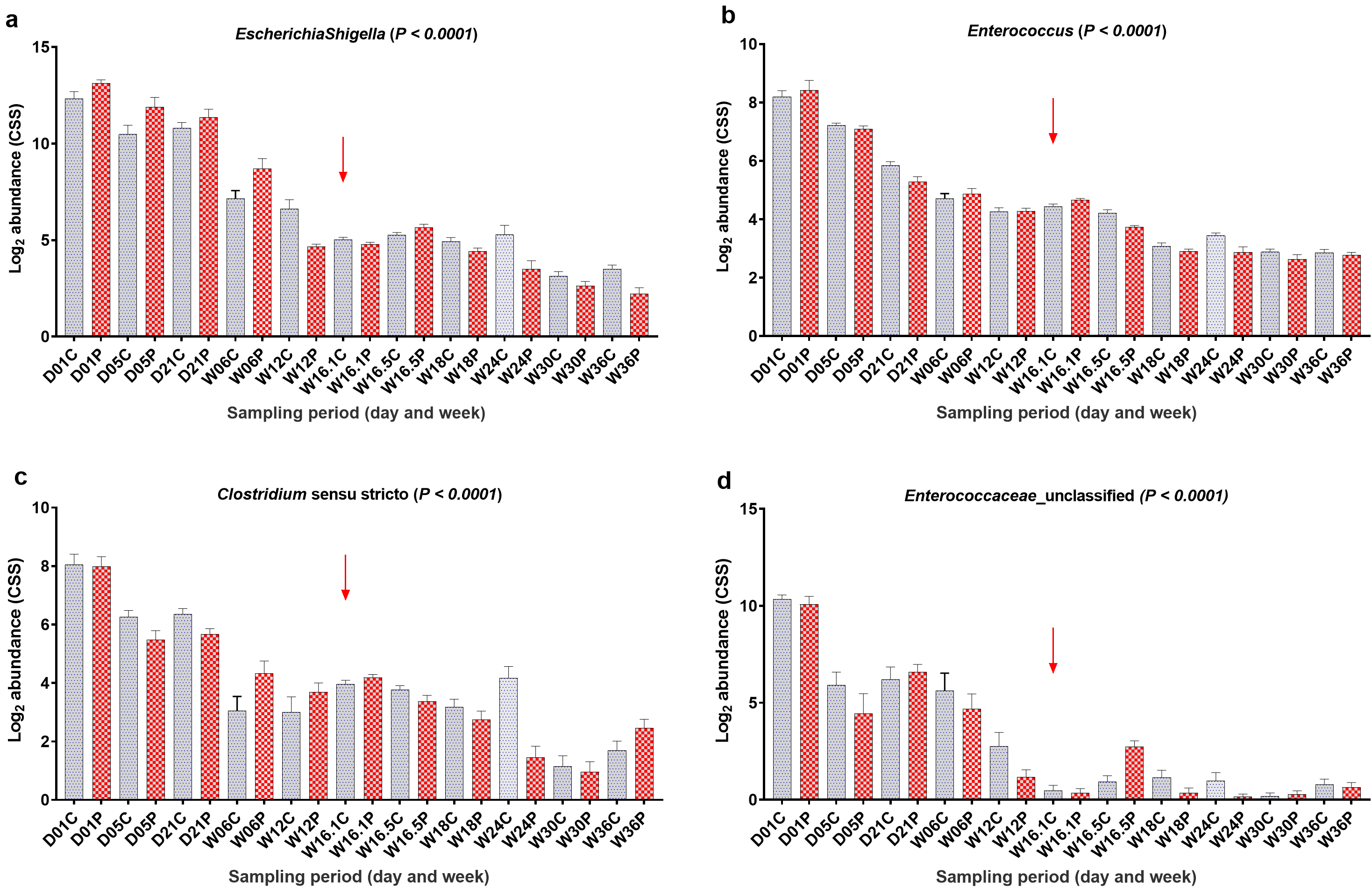

Supplement: Supplementary file 8 — Additional file 8 Fig. S4. Abundance of gut microbiota at genus level affected by probiotic supplementation at multiple sampling periods. a). EscherichiaShigella. b). Enterococcus. c). Clostridium sensu stricto. d). Enterococcaceae_unclassified. In each panel of the Supplementary Fig. S4, the letters “D” and “W” refer to day and week post-hatch, while the letters “C” and “P” refer to control and probiotic supplemented groups, respectively. [file 42523_2021_112_MOESM8_ESM.tif]

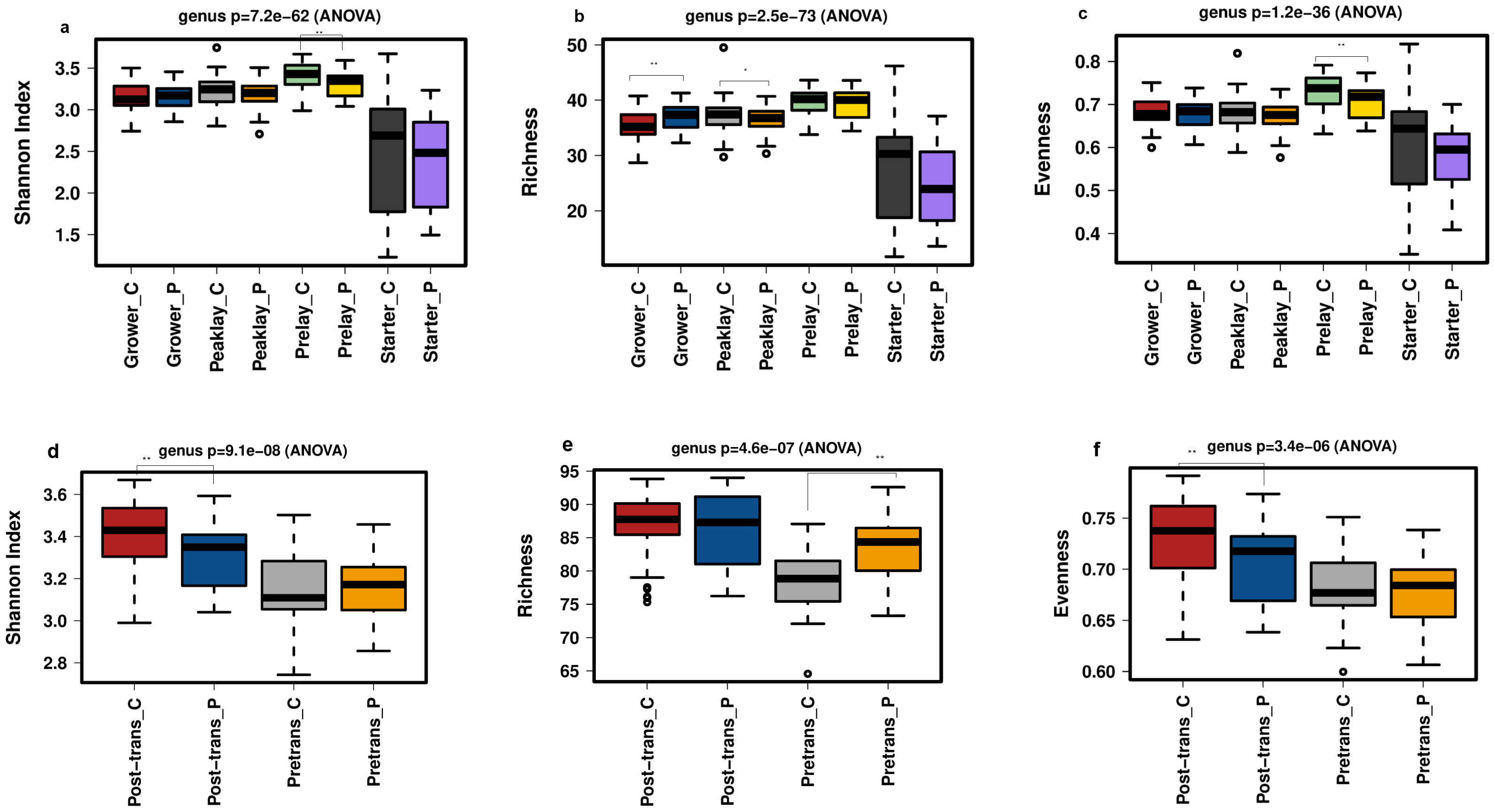

Supplement: Supplementary file 9 — Additional file 9 Fig. S5. Alpha diversity of gut microbiota of laying chickens affected by probiotic supplementation, rearing and laying conditions. a). Alpha diversity (Shannon index) of gut microbiota affected by feeding regimen. b). Richness of gut microbiota affected by feeding regimen. c). Evenness of gut microbiota affected by feeding regimen. d). Alpha diversity (Shannon index) of gut microbiota affected by transport stress. e). Richness of gut microbiota affected by transport stress. f). Evenness of gut microbiota affected by transport stress. Within each treatment group, “C” refers to control, while “P” refers to the probiotic supplemented cohort. Alpha diversity was measured at genus level by Shannon index, Richness and Evenness in Calypso software. Within each treatment group at specific sampling period, asterisk (*) shows a significant difference at P < 0.01, while asterisks (**) show P < 0.001. [file 42523_2021_112_MOESM9_ESM.tif]

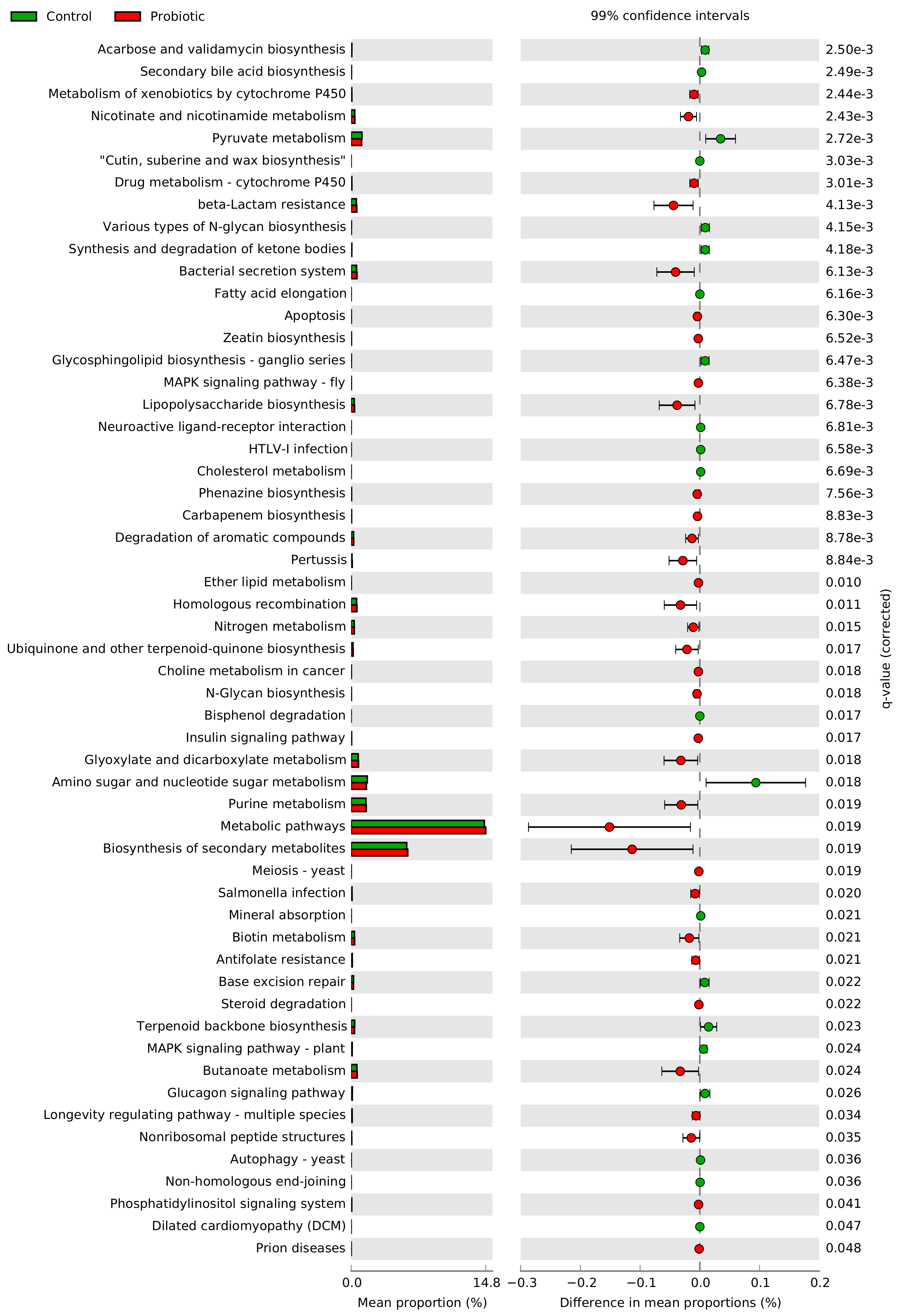

Supplement: Supplementary file 11 — Additional file 11 Fig. S6. Metabolic pathways of the gut microbiota affected by probiotic supplementation. To understand the effect of Bacillus based probiotic on the differential abundance of metabolic pathways of the gut microbiota, the functional prediction data obtained through Tax4Fun2 were analysed in STAMP by using Welch’s t test with 99% confidence interval. For multiple test correction in STAMP, Benjamini-Hochberg was used with q value filter > 0.05 that resulted only in the features that were significantly different between the two treatment groups. [file 42523_2021_112_MOESM11_ESM.tif]

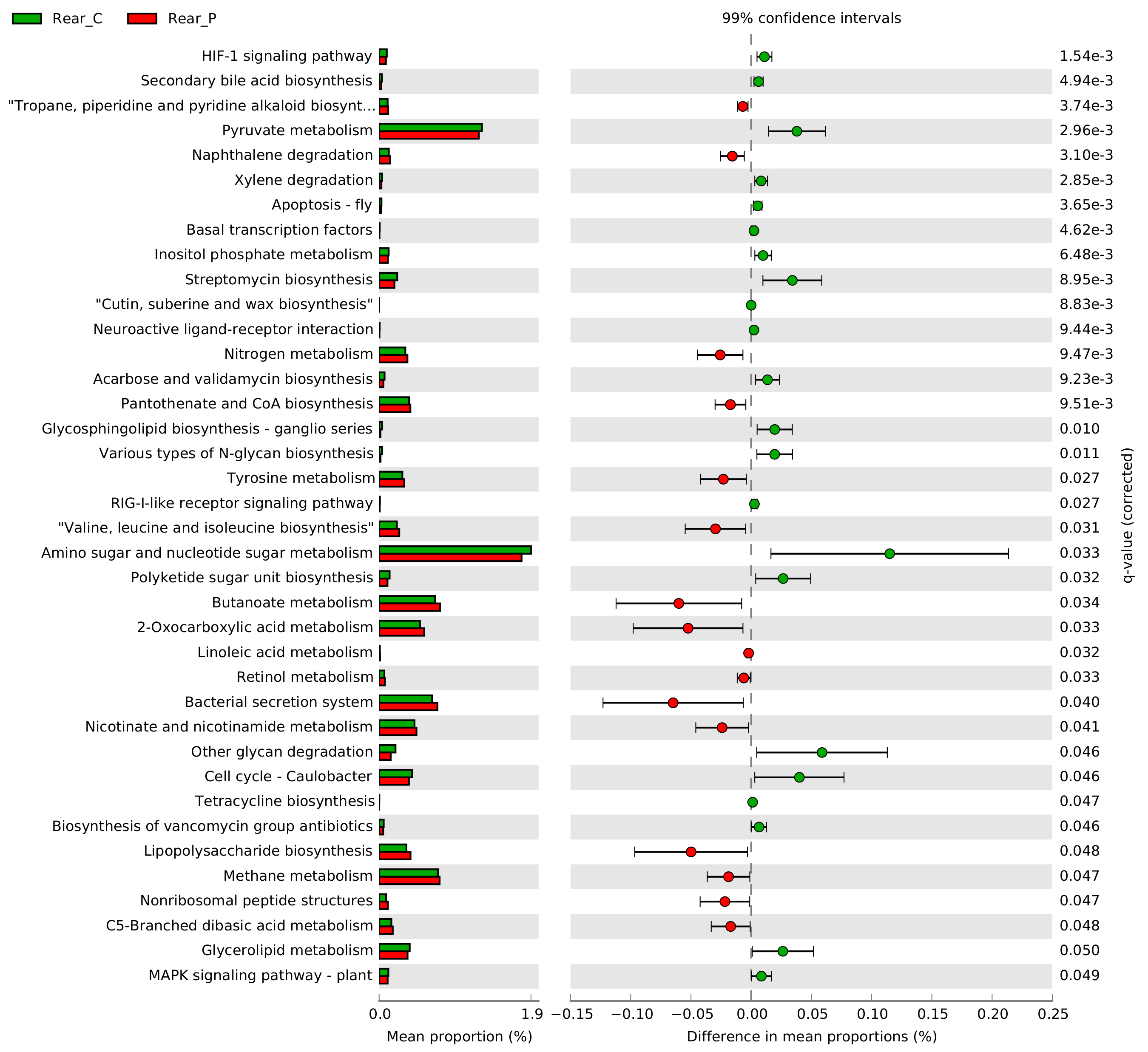

Supplement: Supplementary file 12 — Additional file 12 Fig. S7. Metabolic pathways of the gut microbiota affected by probiotic supplementation in the rearing phase of laying flock. To understand the effect of Bacillus based probiotic on the differential abundance of metabolic pathways of the gut microbiota, the functional prediction data obtained through Tax4Fun2 were analysed in STAMP by using Welch’s t test with 99% confidence interval. For multiple test correction in STAMP, Benjamini-Hochberg was used with q value filter > 0.05 that resulted only in the features that were significantly different between the two treatment groups. In the figure, titles Rear_C and Rear_P refer to faecal samples collected from the control and probiotic supplemented cohorts at rearing phase of flock age, respectively. [file 42523_2021_112_MOESM12_ESM.tif]

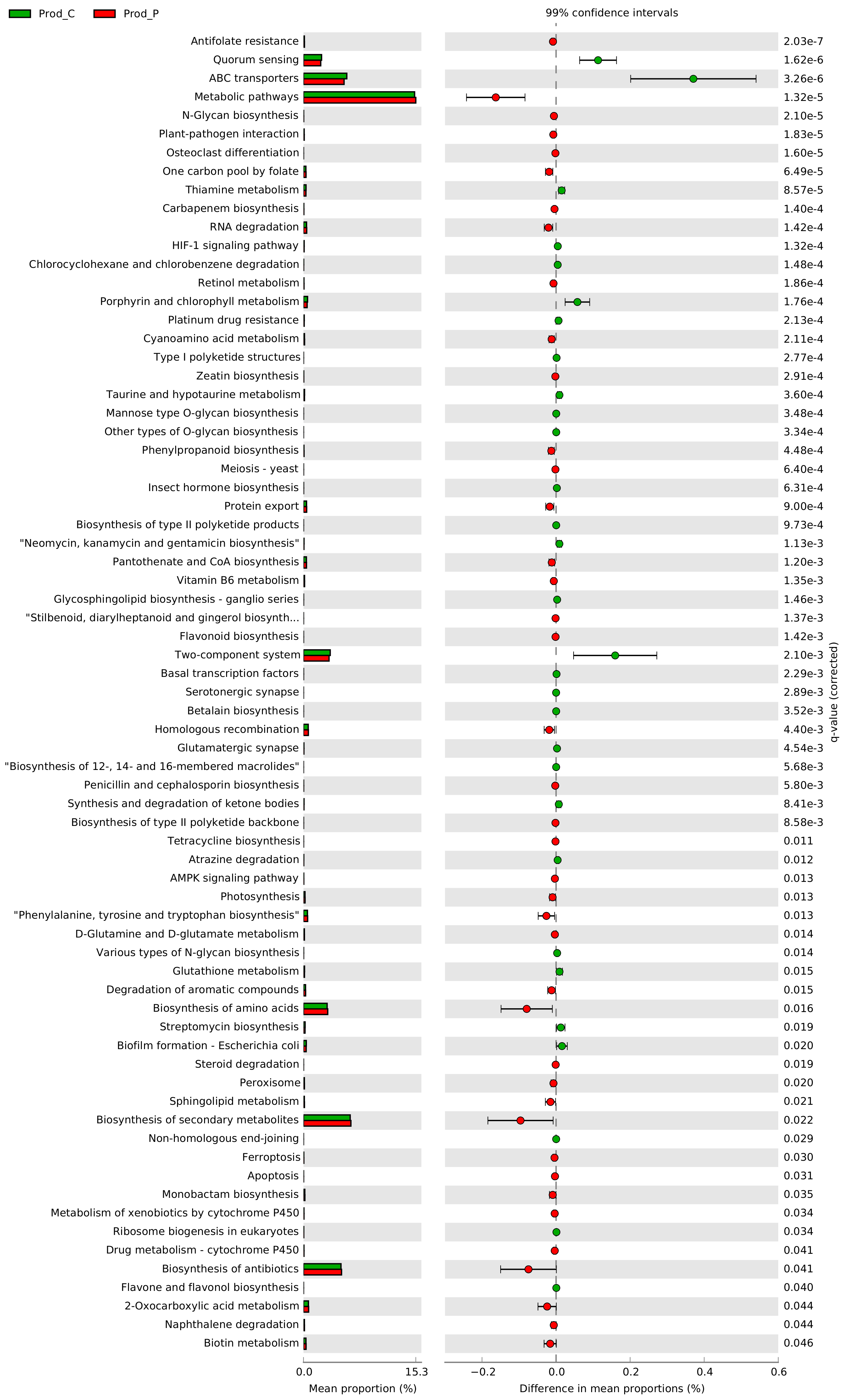

Supplement: Supplementary file 13 — Additional file 13 Fig. S8. Metabolic pathways of the gut microbiota affected by probiotic supplementation in the rearing phase of laying flock. To understand the effect of Bacillus based probiotic on the differential abundance of metabolic pathways of the gut microbiota, the functional prediction data obtained through Tax4Fun2 were analysed in STAMP by using Welch’s t test with 99% confidence interval. For multiple test correction in STAMP, Benjamini-Hochberg was used with q value filter > 0.05 that resulted only in the features that were significantly different between the two treatment groups. In the figure, titles Prod_C and Prod_P refer to faecal samples collected from the control and probiotic supplemented cohorts at production phase of flock age, respectively. [file 42523_2021_112_MOESM13_ESM.tif]

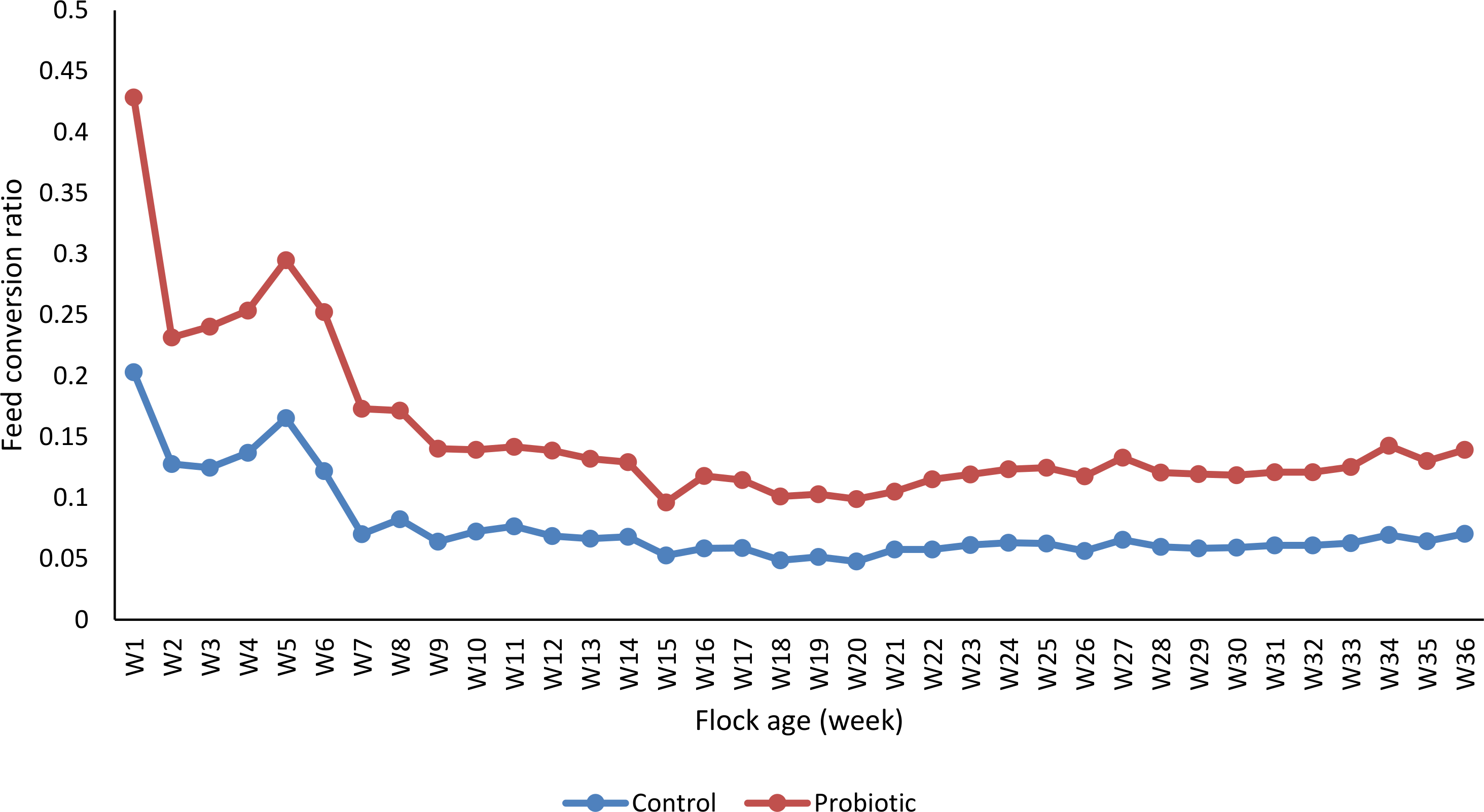

Supplement: Supplementary file 14 — Additional file 14 Fig. S9. Feed conversion ratio of the probiotic supplemented and control flocks. Weekly (W) feed conversion ratio was calculated from feed intake and flock body weight. [file 42523_2021_112_MOESM14_ESM.tif]

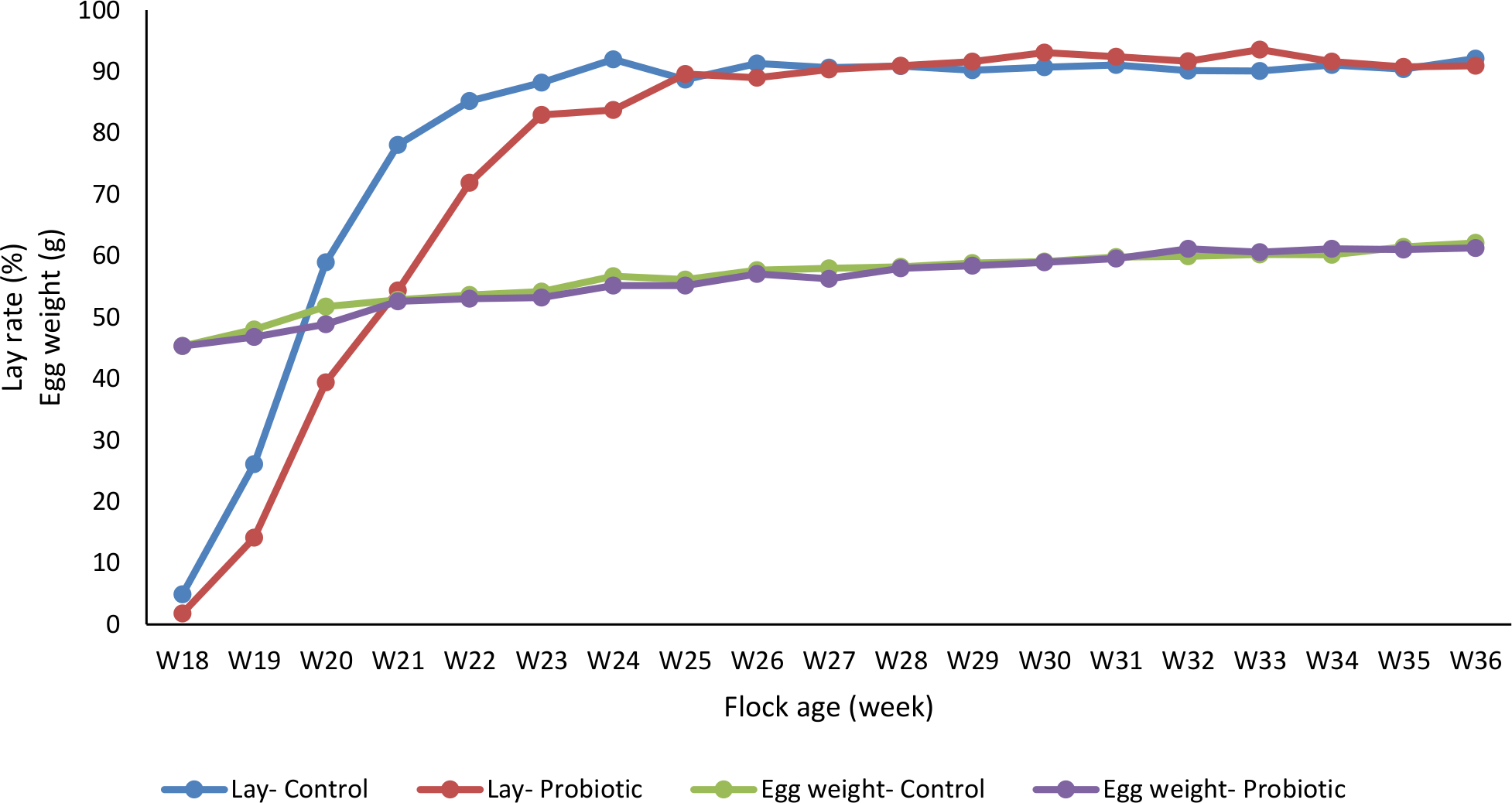

Supplement: Supplementary file 15 — Additional file 15 Fig. S10. Lay rate and egg weight of probiotic supplemented and control flocks. [file 42523_2021_112_MOESM15_ESM.tif]

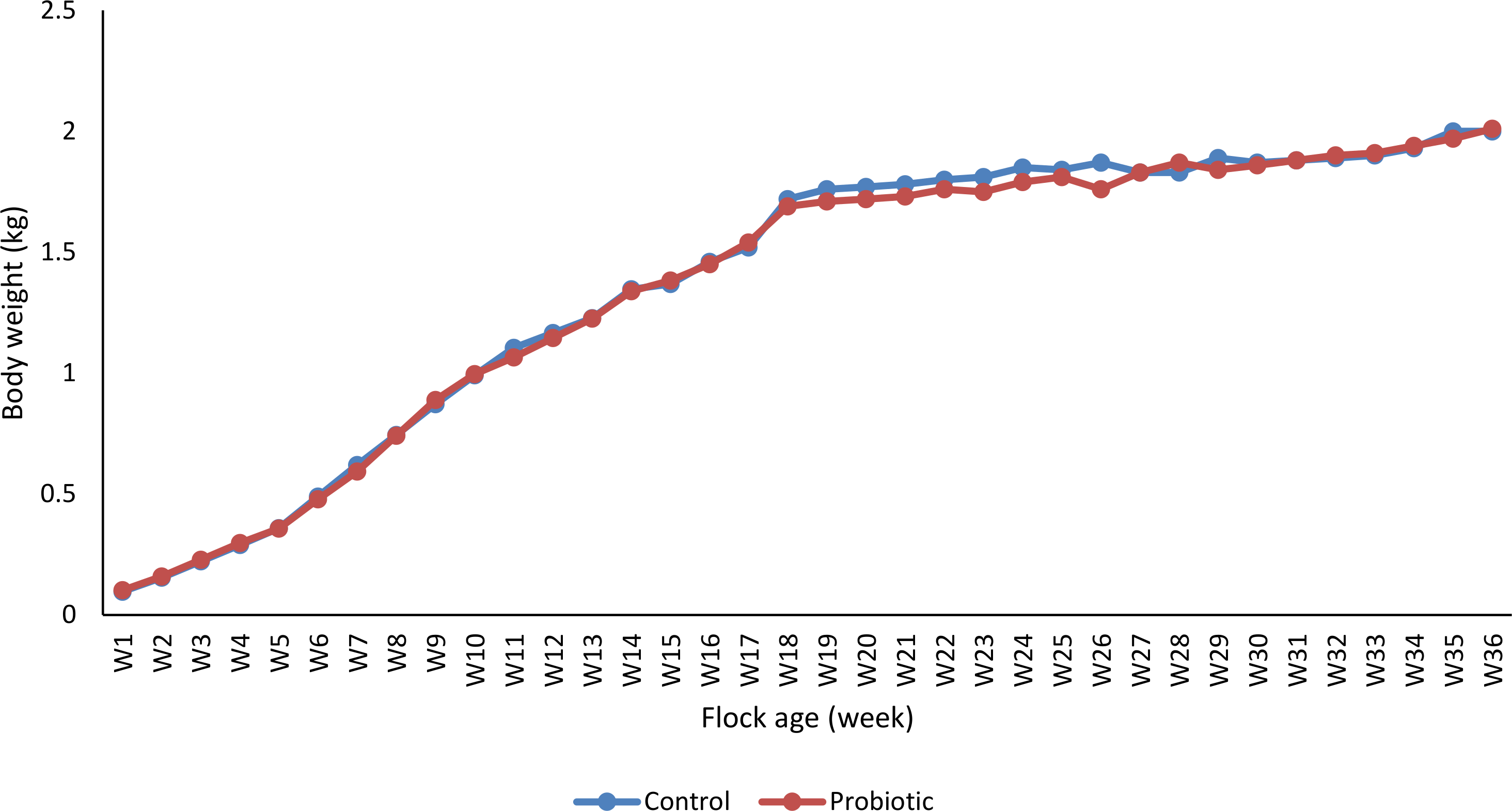

Supplement: Supplementary file 16 — Additional file 16 Fig. S11. Body weight of probiotic supplemented and control flocks. Body weight of 100 randomly selected chickens was taken weekly (W) from each of the probiotic supplemented and control flocks. [file 42523_2021_112_MOESM16_ESM.tif]

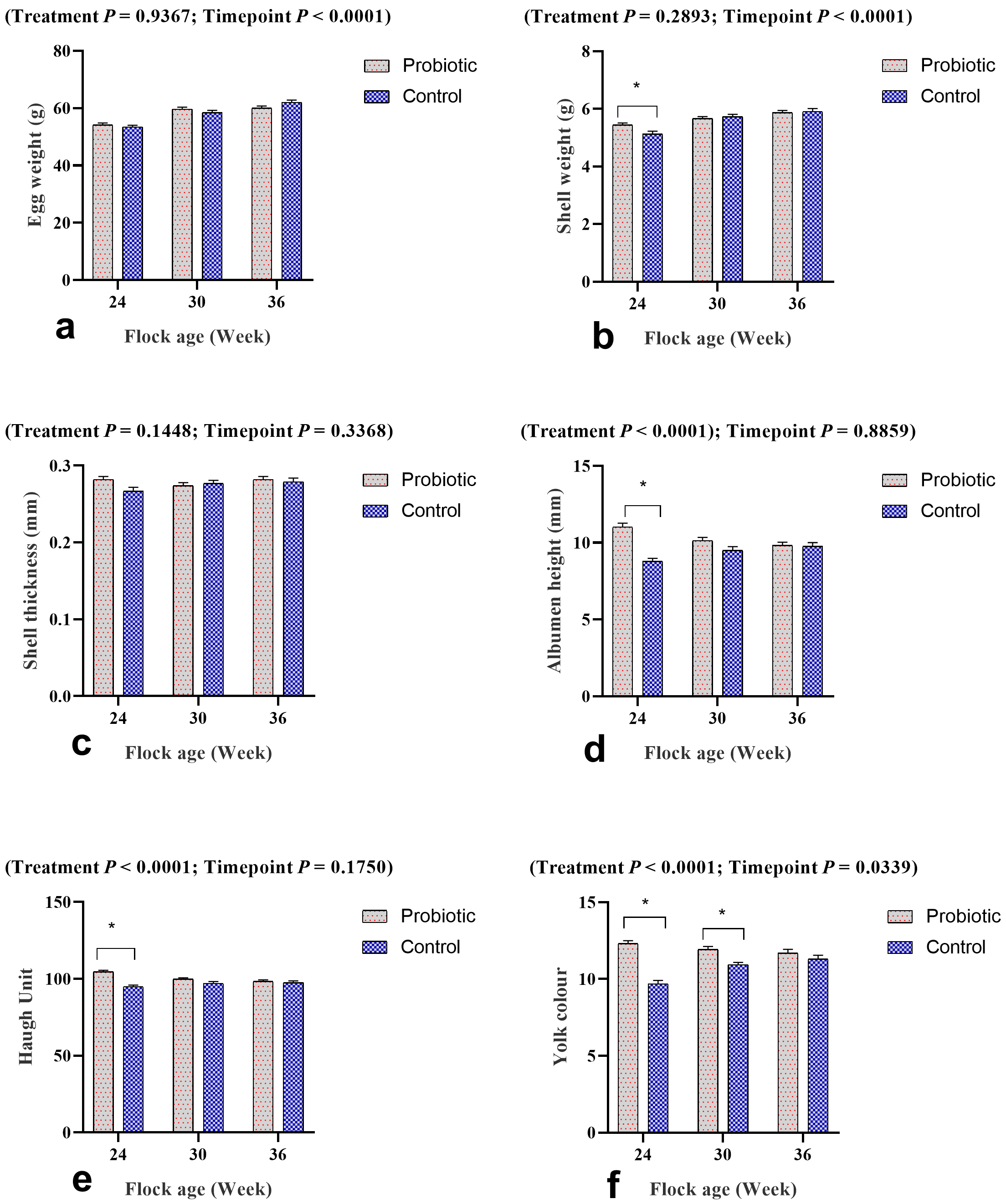

Supplement: Supplementary file 17 — Additional file 17 Fig. S12. Egg quality parameters of eggs collected from probiotic supplemented and control cohorts. a) Egg weight (g). b) Shell weight (g). c) Shell thickness (mm). d) Albumen height (mm). e) Haugh Unit. f) Yolk colour. Freshly laid eggs were collected at week 24, 30 and 36 week of flock age and immediately analysed for egg shell and internal quality parameters. [file 42523_2021_112_MOESM17_ESM.tif]

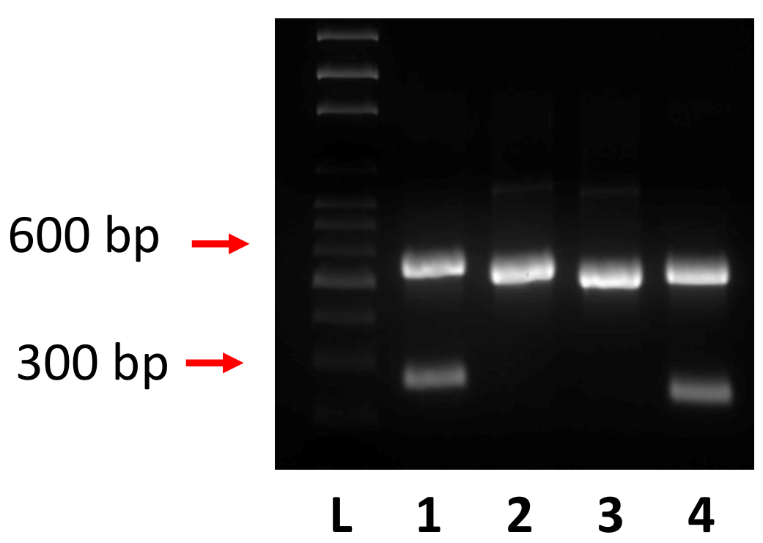

Supplement: Supplementary file 18 — Additional file 18 Fig. S13. PCR products of Salmonella positive samples visualised on 2% agarose gel. L) Ladder; 1) Salmonella Typhimurium isolated from faecal swab at week 18 of flock age from the control shed. 2) Salmonella spp. isolated from environmental swab isolated at week 36 of flock age from the control shed; 3) Salmonella spp. isolated from environmental swab isolated at week 36 of flock age from the control shed. 4) Salmonella Typhimurium as positive control. For Salmonella spp. typing primer pair (605 bp) amplifying the fragment of invA gene were used, while for Salmonella Typhimurium confirmation, primer pair (303 bp) amplifying the fragment of TSR3 gene were used. [file 42523_2021_112_MOESM18_ESM.tif]
